# An endoplasmic reticulum stress-related signature could robustly predict prognosis and closely associate with response to immunotherapy in pancreatic ductal adenocarcinoma

**DOI:** 10.1007/s00432-023-05312-x

**Published:** 2023-08-31

**Authors:** Shuguang Liu, Qianying Hu, Zishan Xie, Shaojing Chen, Yixuan Li, Nali Quan, Kaimeng Huang, Riqing Li, Lishan Fang

**Affiliations:** 1 Department of Pathology, The Eighth Affiliated Hospital, Sun Yat-Sun University, Shenzhen, 518033 China; 2 Medical Research Center, The Eighth Affiliated Hospital, Sun Yat-Sun University, Shenzhen, 518033 China; 3 Department of Breast Surgery, The Eighth Affiliated Hospital, Sun Yat-Sun University, Shenzhen, 518033 China; 4 Clinical Laboratory, The Eighth Affiliated Hospital, Sun Yat-Sun University, Shenzhen, 518033 China; 5 Division of Radiation and Genome Stability, Department of Radiation Oncology, Dana-Farber Cancer Institute, Harvard Medical School, Boston, MA 02215 USA; 6 CAS Key Laboratory of Regenerative Biology, Guangzhou Institutes of Biomedicine and Health, Chinese Academy of Sciences, Guangzhou, 510530 China; 7 Shenzhen Agricultural Technology Promotion Center, Shenzhen, 518005 China

**Keywords:** Pancreatic ductal adenocarcinoma, Overall survival, Endoplasmic reticulum stress, Prognosis, Immunotherapy response

## Abstract

**Purpose:**

Pancreatic ductal adenocarcinoma (PDAC) is one of the most malignant tumors. Endoplasmic reticulum stress (ERS) plays an essential role in PDAC progression. Here, we aim to identify the ERS-related genes in PDAC and build reliable risk models for diagnosis, prognosis and immunotherapy response of PDAC patients as well as investigate the potential mechanism.

**Methods:**

We obtained PDAC cohorts with transcriptional profiles and clinical data from the ArrayExpress, The Cancer Genome Atlas (TCGA) and Genotype-Tissue Expression (GTEx) databases. Univariate Cox regression, LASSO regression and multivariate Cox regression analyses were used to construct an ERS-related prognostic signature. The CIBERSORT and ssGSEA algorithms were applied to explore the correlation between the prognostic signature and immune cell infiltration and immune-related pathways. The GDSC database and TIDE algorithm were used to predict responses to chemotherapy and immunotherapy, identifying potential drugs for treating patients with PDAC.

**Results:**

We established and validated an ERS-related prognostic signature comprising eight genes (HMOX1, TGFB1, JSRP1, GAPDH, CAV1, CHRNE, CD74 and ERN2). Patients with higher risk scores displayed worse outcomes than those with lower risk scores. PDAC patients in low-risk groups might benefit from immunotherapy. Dasatinib and lapatinib might have potential therapeutic implications in high-risk PDAC patients.

**Conclusion:**

We established and validated an ERS-related prognostic signature comprising eight genes to predict the overall survival outcome of PDAC patients, which closely correlating with the response to immunotherapy and sensitivity to anti-tumor drugs, as well as could be beneficial for formulating clinical strategies and administering individualized treatments.

**Supplementary Information:**

The online version contains supplementary material available at 10.1007/s00432-023-05312-x.

## Introduction

Pancreatic ductal adenocarcinoma (PDAC) is a malignant tumor accounting for approximately 90% of all pancreatic cancers with an average 5-year survival rate of only 10% (Siegel et al. 2021). The poor prognosis of PDAC is mostly due to the lack of effective early diagnostic and screening methods. Due to the biological heterogeneities that exist among PDAC patients, treatment should be individualized and systemic to prolong survival (Allen et al. 2017; van Roessel et al. 2018). Although the American Joint Committee on Cancer (AJCC) TNM staging is presently the most widely used traditional risk stratification system, it still has limitations in predicting prognosis and treatment sensitivity. As a result, building an accurate prognostic prediction model for PDAC patients is critical.

The endoplasmic reticulum (ER) is a specialized organelle that regulates protein synthesis, folding, secretion and posttranslational modification. Cells respond to tumor microenvironment stimuli, protein misfolding occurs, and misfolded proteins accumulate in the ER, which is referred to as ERS. Subsequently, the ER triggers the unfolded protein response to reinstate ER homeostasis and support adaptation to various changes in the tumor. Previous studies have reported increased ERS in several types of malignant cells, including those from patients with breast cancer, squamous carcinoma, and PDAC (Ranganathan et al. 2006; Bartkowiak et al. 2010; Pommier et al. 2018). Prolonged ERS is conducive to organelle damage and dysfunction and leads to cell death (Wang and Kaufman 2014; Robinson et al. 2021), while modulation of ERS could induce tumor dormancy or promote the immunosuppressive ability of tumor-infiltrating myeloid-derived suppressor cells to promote tumor development (Lee et al. 2014; Garcia-Carbonero et al. 2018). However, the role of ERS during PDAC progression remains to be uncovered. Therefore, a thorough understanding is needed to develop better treatments for PDAC patients.

In this study, we established an ERS-related prognostic risk model based on a pancreatic cancer cohort from the E-MTAB-6134, The Cancer Genome Atlas (TCGA) databases and Gene Expression Omnibus (GEO). The ERS-related prognostic signature was established by applying univariate Cox and least absolute shrinkage and selection (LASSO) regression analyses; the model was then verified in the external dataset. We also integrated the ERS-related signature and clinical characteristics to develop a nomogram to predict the overall survival (OS) outcomes of PDAC patients. Moreover, we assessed the effectiveness of ERS-related prognostic model in determining patients to receive immunotherapy and the sensitivity of anti-tumor drugs. In summary, the ERS-related prognostic risk model has important potential to predict the prognosis of PDAC patients and assist in clinical decision-making.

## Material and methods

### Study design and data acquisition

**Fig. 1:**
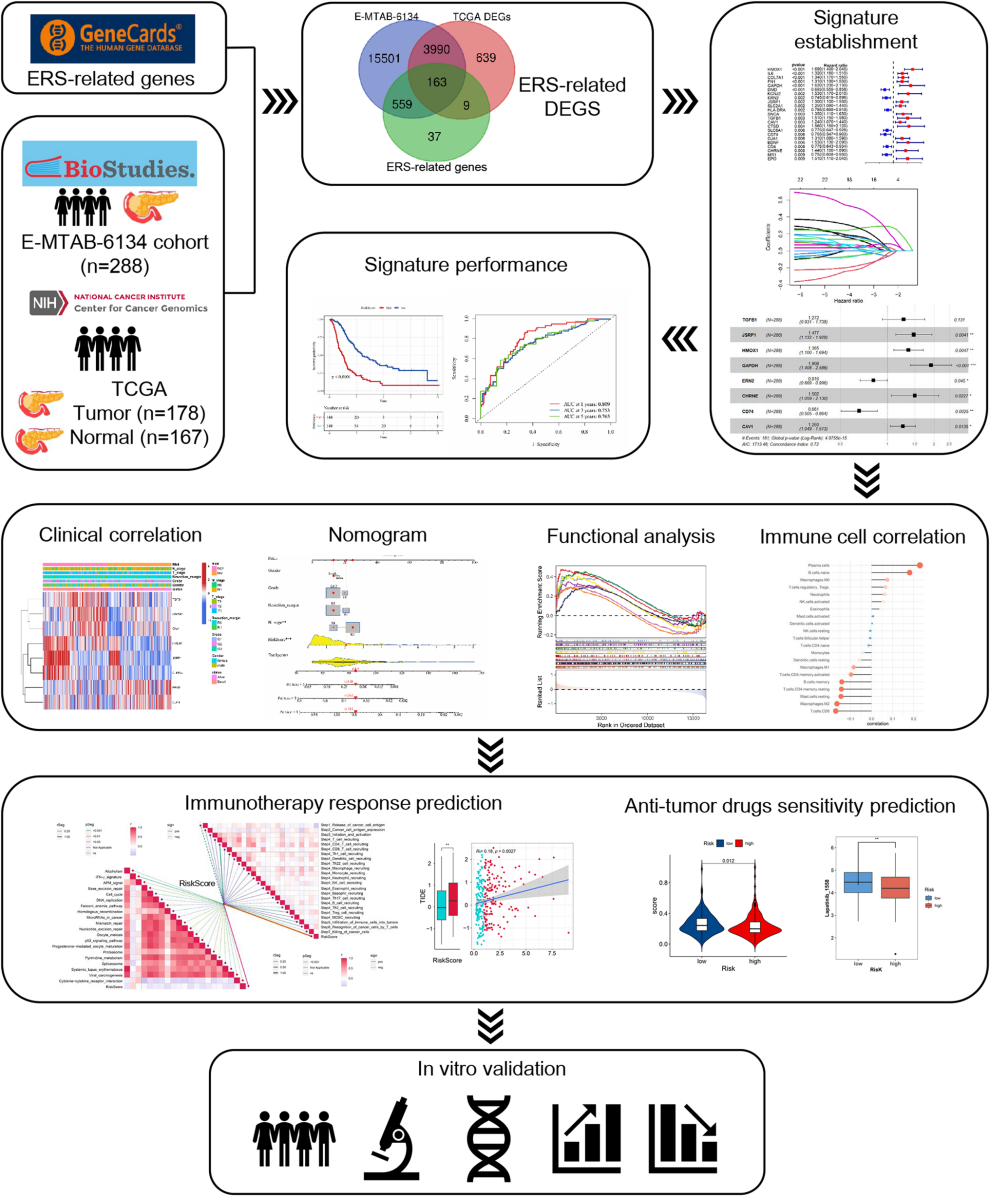
Flowchart of the study

Figure 1 depicts our analysis workflow. First, we obtained 768 ERS-related genes from GeneCards (https://www.genecards.org/). The E-MTAB-6134 cohort (n = 288) from the ArrayExpress database (https://www.ebi.ac.uk/biostudies/arrayexpress) included in this study for survival analyses was used as the training set. The RNA-seq data and clinical information of 178 pancreatic cancer samples and 167 normal samples in TCGA and Genotype-Tissue Expression (GTEx) were downloaded from the UCSC Xena Database (https://xenabrowser.net/datapages/). The GSE21501 (n = 102), GSE28735 (n = 42) and GSE62452 dataset (n = 66) from Gene Expression Omnibus (GEO, http://www.ncbi.nlm.nih.gov/geo) were retrieved and used as a validation set to validate the ERS-related signature. GSE41368 and GSE62165 dataset were used to investigate the expression of ERS-related gene in tumor and normal tissues of PDAC patients.

### Identification of the differentially expressed ERS-related genes

To identify the differentially expressed genes (DEGs) among the 768 ERS-related genes, we normalized the raw read counts and screened out DEGs between tumor and normal tissues in the TCGA-PDAC cohort by the R package “DEseq2”, using the criteria |log2-fold change (FC)| > 1.5 and false discovery rate (FDR) < 0.05 (Table S1). The protein–protein interaction (PPI) network for DEGs were analyzed by R package “igraph” and the Search Tool for the Retrieval of Interacting Genes (STRING, https://cn.string-db.org/).

### Functional enrichment analysis of the differentially expressed ERS-related genes

To identify the relevant pathways in the ERS-related signature, Gene Ontology (GO, http://www.geneontology.org) and Kyoto Encyclopedia of Genes and Genomes (KEGG, http://www.kegg.jp/) pathway enrichment analyses were conducted via the “clusterProfiler” and “GOplot” R packages. An adjusted *p* value < 0.05 was set as the threshold for statistical significance.

### Construction of a prognostic risk model based on the ERS-related genes

To identify the 163 ERS-related genes associated with prognosis in PDAC patients, univariate Cox regression analysis was performed (*p* < 0.01). LASSO regression analysis was applied to reduce the dimensionality of candidate genes according to the best penalty factor (λ). Then, the prognostic ERS-related genes were used to construct the ERS-related signature, and the risk scores were generated in a multivariate Cox regression model with the following formula: RiskScore = ${\sum }_{i=1}^{n}(\text{expression of signature gene i} \times \text{coefficient } \upbeta \text{ i})$

### Evaluation of the prognostic accuracy of the ERS-related signature

The patients were divided into low-risk and high-risk groups according to the median risk score. Survival curves were generated using the Kaplan‒Meier method with a log-rank *p* value < 0.05 for the survival difference between the two groups. Receiver operator characteristic (ROC) curves and area under the curve (AUC) values corresponding to 1, 3, and 5 years were used to evaluate the predictive power of the prognostic signature. Then, we performed a correlation analysis to verify the connections between the risk score and patient clinicopathological characteristics. Univariate and multivariate Cox regression analyses were conducted to validate the independent prediction ability of the gene signature. In addition, a stratified analysis was utilized to examine the precision of prognostic prediction based on other clinical pathological features.

### Construction and evaluation of the nomogram

Considering both clinical information and the risk score, a nomogram was drawn with the relevant R package to predict the 1-, 3-, and 5-year survival rates. The predictive performance of the nomogram was identified using the consistency index and calibration curves. The “ggDCA” R package was used to perform decision curve analysis (DCA) to verify the accuracy of the prognostic signature.

### Gene set enrichment analysis

Gene set enrichment analysis (GSEA) was conducted to identify the pathways that were significantly different between the ERS-related high- and low-risk groups. The risk score was used as the phenotype label. The nominal *p* value and normalized enrichment score (NES) were evaluated to sort the pathways enriched in each phenotype. Gene set permutations for each analysis were executed 1000 times. An absolute value of the standardized NES > 1 and a nominal *p* value less than 0.05 were regarded as the threshold of statistical significance (Table S2).

### Immune infiltration analysis of the ERS-related signature

The immune infiltration scores were measured by TIMER, CIBERSORT, xCell and ssGSEA. Spearman correlation analysis was applied to evaluated the correlation between risk score and immune cells. ssGSEA was applied to calculate the scores of infiltrating immune cells and to evaluate the activity of immune-related pathways. Wilcoxon test was carried out to assess the correlation between the ERS-related risk score and the expression of 40 immune checkpoint genes.

### Correlation between immunotherapy response and the ERS-related signature

The immunotherapeutic signatures and the activity of each step of the cancer-immunity cycle was evaluated via ssGSEA algorithm. The “ggcor” R package was used to analyze the correlation of risk score with immunotherapeutic signatures and cancer-immunity cycles. The Tumor Immune Dysfunction and Exclusion (TIDE) algorithm was used to predict the immune responses of the ERS-related signature to anti-PD-1 and anti-CTLA-4 treatment (Jiang et al. 2018). The expression levels of the marker of IFN-γ pathway, m6A regulator genes, 148 immunomodulators including chemokines, MHC, receptors, immunoinhibits and immunostimulators in ERS-related risk groups were explored by Wilcoxon test.

### Relationship between the ERS-related signature and chemotherapy

The IC50 was evaluated via the Genomics of Drug Sensitivity in Cancer (GDSC, https://www.cancerrxgene.org) database and the chemotherapy response was predicted with the “oncoPredict” package of R. Six chemotherapy drugs (dasatinib, paclitaxel, BI.2536, gemcitabine, lapatinib and docetaxel) were selected to predict the IC50 values in patients. The Wilcoxon rank-sum test was used to analyze the difference between the two risk groups. Tumor stemness score was analyzed by one-class logistic regression (OCLR) via performing “limma” R package.

### Cell culture

The human PDAC cell line AsPC-1 was purchased from Procell and verified through short tandem repeat (STR) sequence identification. AsPC-1 cell was cultured in RPMI 1640 medium, supplemented with 10% bovine serum, 1% penicillin–streptomycin (Gibco, CA, USA) and maintained at 37 °C in a humidified incubator with 5% CO_2_.

### Formalin-fixed and paraffin-embedded (FFPE) samples acquisition

5 FFPE samples were collected from patients diagnosed with PDAC from January 2021 to December 2022 at the Eighth Affiliated Hospital of Sun Yat-Sen University. The samples of surgically resected cancerous and normal tissues were preserved after formalin fixation and paraffin embedding treatment.

### Transfection of siRNAs

HMOX1 and TGFB1 specific siRNA and negative control siRNA were purchased from GenePharma, Suzhou, China. The siRNAs were transfected into cells using jetPRIME transfection reagent (Polyplus Transfection, Illkirch, France) following the guideline. The sequences of siRNAs are shown in Table S3.

### RNA isolation and quantitative PCR (q-PCR) assay

Total RNA was extracted from cells using Total RNA Kit II (Genebase Bioscience, China). Total RNA extracted from FFPE tissues was using FFPE RNA Kit (Genebase Bioscience, China) following the manufacturer’s instruction. q-PCR assay was performed using SYBR Green Pro Taq HS Premix (Accurate Biology, Shenzhen, China) and carried out in the LightCycler 480 (Roche, Basel, Switzerland). The relevant primers used are shown in Table S4.

### Cell viability

Cell viability was measured using Cell Counting Kit-8 (CCK-8, Dojindo, Kumamoto, Japan) following the manufacturer’s instructions. Cells after indicated treatment were seeded in 96-well plates at 5000 cells/well and cultured. Then, the CCK-8 solution (10μL) was added to each well at 48 h. Next, 1.5 h after CCK-8 administration, the absorbance was measured at 450 nm wavelength with spectrophotometer.

### Colony formation assay

Cells after indicated treatment were reseeded in 12-well plates at density of 500 cells/well and cultured for 2 weeks. Then fixed with 4% paraformaldehyde, stained with crystal violet and counted the number of the colonies formed to quantification.

### Library construction and sequencing

RNA was harvested using Rneasy mini plus kit (Qiagen). 1.4 ug of total RNA was used for the construction of sequencing libraries. After total RNA was extracted, RNA libraries for RNA-seq were prepared using Hieff NGS™ MaxUp Dual-mode mRNA Library Prep Kit for Illumina^®^ following manufacturer's protocols. The resulting library was sequenced using DNBSEQ-T7 by Sangon Biotech Co. (Shanghai, China).

### Statistical analysis

All statistical analyses were conducted with the R and R Bioconductor packages. Kaplan–Meier curves were drawn by the “survival” package, and the differences were evaluated by the log-rank test. Univariate and multivariate Cox regression analyses were applied for independent prognostic analysis. ROC analysis was used to detect the sensitivity and specificity of the risk score in predicting survival. The AUC can be used as an index of prognostic accuracy. Spearman correlation analysis was used to evaluated the relevance between risk score and infiltrated immune cells. In all analyses, quantification values were presented as mean ± SD, *p* < 0.05 indicated a statistically significant difference.

## Results

### Selection of differentially expressed ERS-related genes

**Fig. 2:**
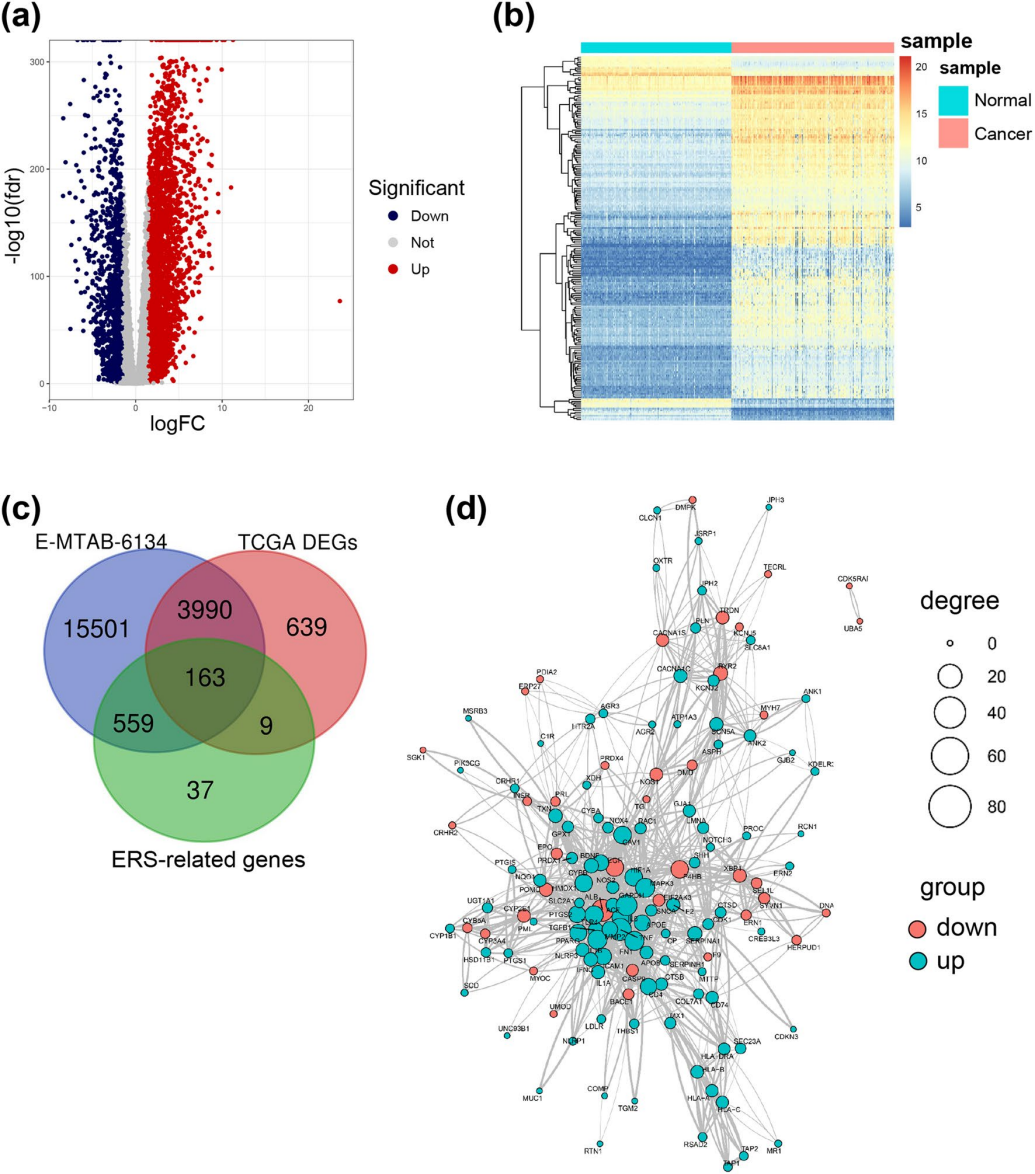
Differentially expressed ERS-related genes of PDAC and normal tissue in TCGA dataset. **a** Volcano presented differentially expressed ERS-related genes. Red showed the upregulation and blue showed the downregulation of ERS-related genes. **b** Heatmap showed the top 200 differentially expression of ERS-related genes. **c** Venn diagram showed the intersect ERS-related genes. **d** The interactions among candidate genes are shown by the PPI network

As outlined in Fig. 2a, we obtained 4801 DEGs (Fig. 2b) by analyzing TCGA data between PDAC tumor and normal samples. We overlapped these 4801 DEGs with 768 ERS-related genes from the GeneCards database and 20,213 genes from the E-MTAB-6134 training set. Subsequently, 163 differentially expressed ERS-related genes from the intersection of above 3 gene lists were selected (Fig. 2c). Among them, 116 genes were upregulated and 47 genes were downregulated. Finally, we uploaded ERS-related DEGs to the STRING website with combined score > 0.5 to explore the interaction of the potential protein (Fig. 2d).

### Functional enrichment analysis

To investigate the functional annotations of the 163 DEGs, we performed GO and KEGG enrichment analyses. The results demonstrated that terms such as calcium ion transport and response to endoplasmic reticulum stress were notably enriched, consistent with the characteristics of ERS-related genes (Fig. S1A). Simultaneously, KEGG pathway enrichment analysis indicated that the DEGs were involved in the phagosome and HIF-1 signaling pathways (Fig. S1B).

### Construction of the ERS-related prognostic signature

**Fig. 3:**
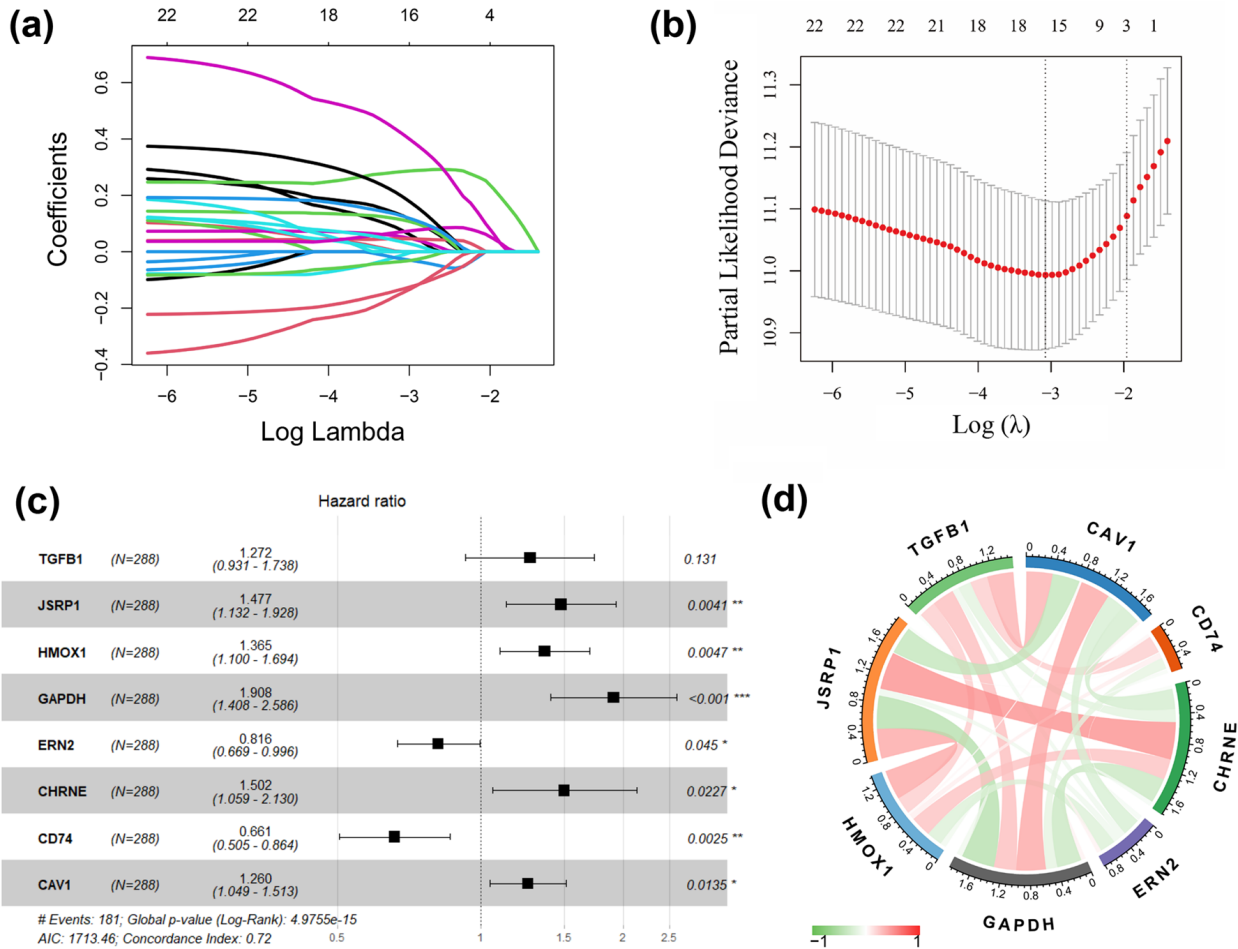
Construction of the ERS-related prognostic signature. **a** 23 ERS-related genes were penalized by LASSO regression analysis. **b** Cross-validation of candidate genes based on the minimum lambda value. **c** Multivariate Cox regression analysis of ERS-related genes. **d** Relevance analysis heat map of 8 ERS-related prognostic genes. *p < 0.05, **p < 0.01, ***p < 0.001

E-MTAB-6134 was used as the training dataset to develop the prognostic signature. First, 23 of the above 163 differentially expressed ERS-related genes were found to be closely related to the prognosis of PDAC patients by univariate Cox regression analysis. Among them, 16 genes were correlated with increased risk with hazard ratios (HRs) > 1; the remaining 7 genes were considered protective with HRs < 1 (Fig. S2). Subsequently, LASSO regression analysis was applied to those 23 genes based on the optimum λ value and 16 candidate genes were screened out (Fig. 3a, b). Finally, by performing multivariate Cox regression analysis, a prognostic model comprising eight genes, HMOX1, TGFB1, JSRP1, GAPDH, ERN2, CHRNE, CD74 and CAV1, was generated to predict the survival outcomes of PDAC patients (Fig. 3c). In addition, eight genes included in ERS-related signature were significantly correlated with each other (Fig. 3d).

### Prognostic performance of the ERS-related signature in the training set and validation sets

**Fig. 4:**
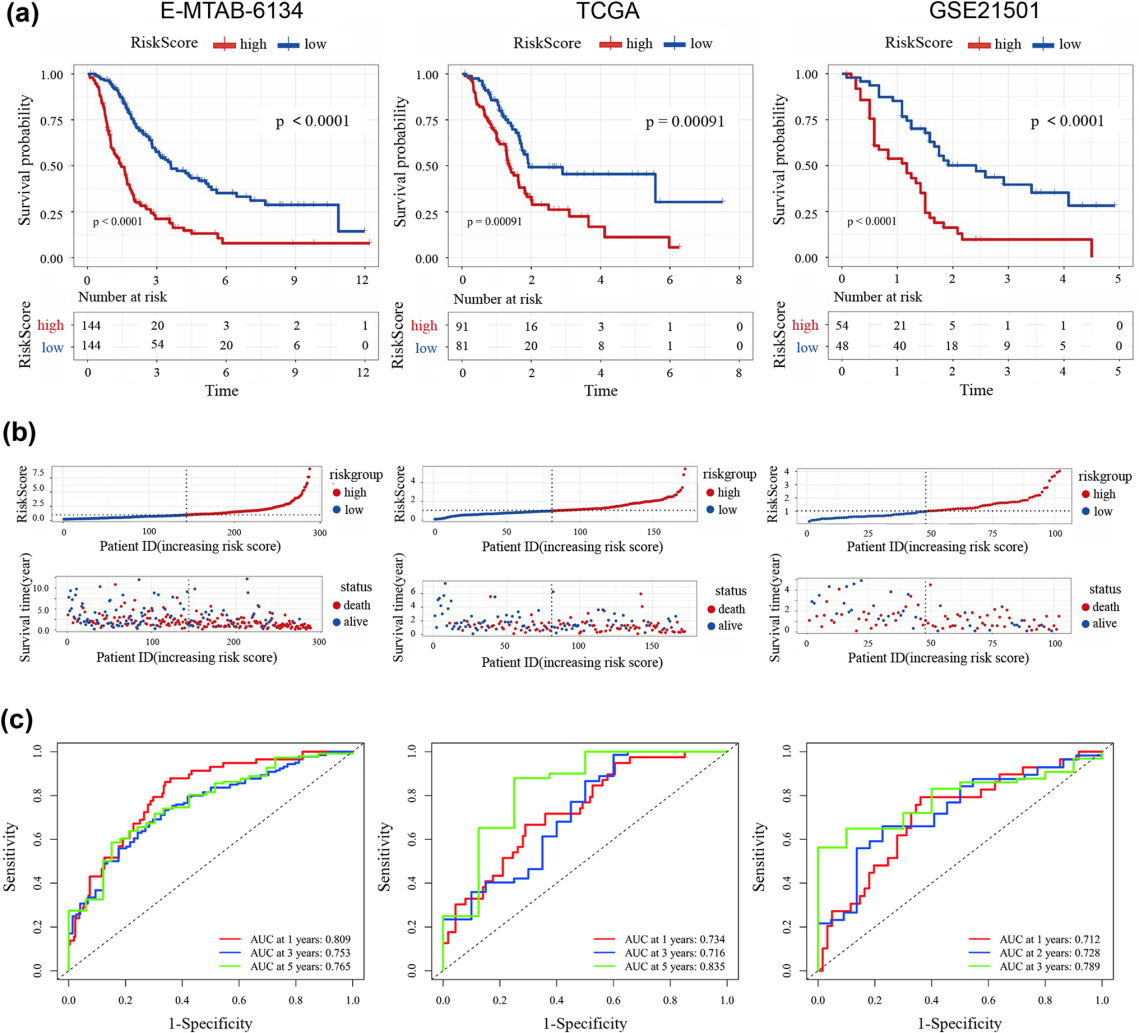
Evaluation and validation of the ERS-related prognostic signature in the training and validation cohorts of PDAC. **a** Kaplan–Meier survival analysis of PDAC patients between high-risk groups and low-risk groups. **b** Distribution of survival status based on the median risk score of PDAC patients. **c** ROC curves to predict the sensitivity and specificity of 1-, 3- and 5-year survival according to the ERS-related signature in training set E-MTAB-6134 and validating set TCGA, while the sensitivity of 1-, 2- and 3-year survival were validated in GSE21501

The risk score was calculated as follows: Risk score = 0.240632 × TGFB1 expression + 0.390215 × JSRP1 expression + 0.311506 × HMOX1 expression + 0.645877 × GAPDH expression + 0.406487 × CHRNE expression + 0.231012 × CAV1 expression − 0.41471 × CD74 expression − 0.20355 × ERN2 expression. ERS-related risk scores for PDAC patients in the training and validation datasets were calculated using the above formula. The median risk score of the training dataset was 0.985, which was considered as the cut-point for dichotomizing the samples into high- and low-risk groups in the training set and validation sets. The distribution of patients was significantly different between the high-and low-risk groups, indicating that the risk score had a powerful discriminatory ability (Fig. S3A). Notably, the Kaplan–Meier survival curves revealed that patients in the high-risk group tended to have worse OS outcomes than those in the low-risk group (Fig. 4a and Fig. S4A). In addition, according to the scatter point analysis, with the increase in the ERS-related risk score, patients tended to have increased mortality rates and reduced survival times (Fig. 4b and Fig. S4B). We also applied the ROC curve to evaluate the predictive role of the risk scores regarding the OS outcome. In the E-MTAB-6134, the AUCs for predicting 1-, 3-, and 5-year OS were 0.809, 0.753 and 0.765, respectively (Fig. 4c). While the AUCs for predicting 1-, 3-, 5-year OS in TCGA were 0.734, 0.716 and 0.835 (Fig. 4c), and 0.755, 0.694 and 0.806 in GSE62452 (Fig. S4C). The AUC values were 0.712 for 1 year, 0.728 for 2 years, 0.789 for 3 years in GSE21501 and 0.768 for 1 year, 0.815 for 2 years, and 0.722 for 3 years in GSE28735. Finally, the heatmap showed that the high-risk group highly expressed HMOX1, TGFB1, JSRP1, GAPDH, CHRNE and CAV1, while ERN2 and CD74 were overexpressed in the low-risk group (Fig. S3B). Taken together, these results demonstrated an excellent predictive performance of the ERS-related prognostic signature.

### Independent prognostic analysis and predictive performance of clinical factors of the ERS-related risk model

**Fig. 5:**
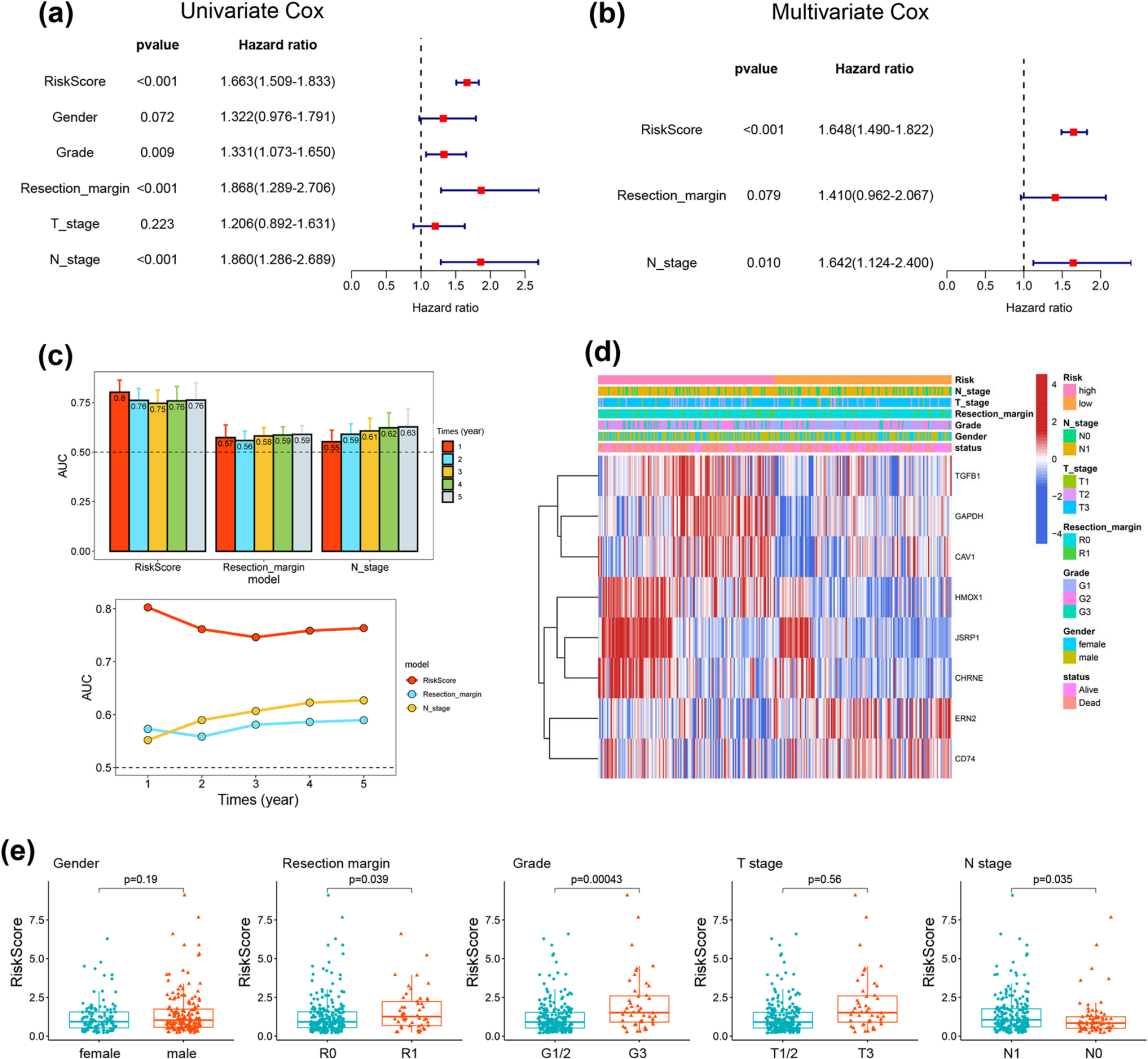
Evaluation of clinical prognostic value of the ERS-related risk model in PDAC. **a**, **b** The correlations between the risk score and clinicopathological factors by univariate and multivariate Cox regression analysis. **c** The AUC values of the clinical characteristics and risk score. **d** Heatmap showed the association of ERS-related signature expression with clinical parameters. **e** The distribution of risk scores by gender, resection margin, tumor grade, and TNM stage

Due to the high correlation between the ERS-related signature and the prognosis of PDAC patients, we evaluated the classic clinical parameters and risk scores by univariate and multivariate Cox regression analyses to identify independent prognostic factors. Through our multivariate Cox regression analysis, grade and resection margin were related to worse survival outcomes, and only N stage and the ERS-related risk score served as independent prognostic factors (Fig. 5a, b). ROC curves were used to evaluate the predictive performance, and the ERS-related risk score with the highest AUC value showed the best discrimination compared with traditional clinicopathological parameters (Fig. 5c). Additionally, the clinical characteristic heatmap showed the tendency of signature gene expression in the two risk groups and were closely correlated with gender, grade, resection margin, T stage and N stage based on the E-MTAB-6134 cohort (Fig. 5d). Next, we investigated the relationship between the ERS-related risk score and clinical characteristics. As shown in Fig. 5e, patients in certain subgroups, such as those with microscopically incomplete resection (R1), a high pathological grade, and an advanced N stage, tended to have higher risk scores, while no differences were obtained with stratification based on gender and T stage. We further confirmed the reliability of the ERS-related prognostic signature. Subgroup analysis revealed that patients with high-risk scores had an unfavorable prognosis compared to those with low-risk scores in the subgroups of gender (male or female), resection margin (R0 or R1), grade (G1/2 or G3), N stage (N0 or N1) and T stage (T1/2 or T3) (Fig. S5).

### Construction of a predictive nomogram

**Fig. 6:**
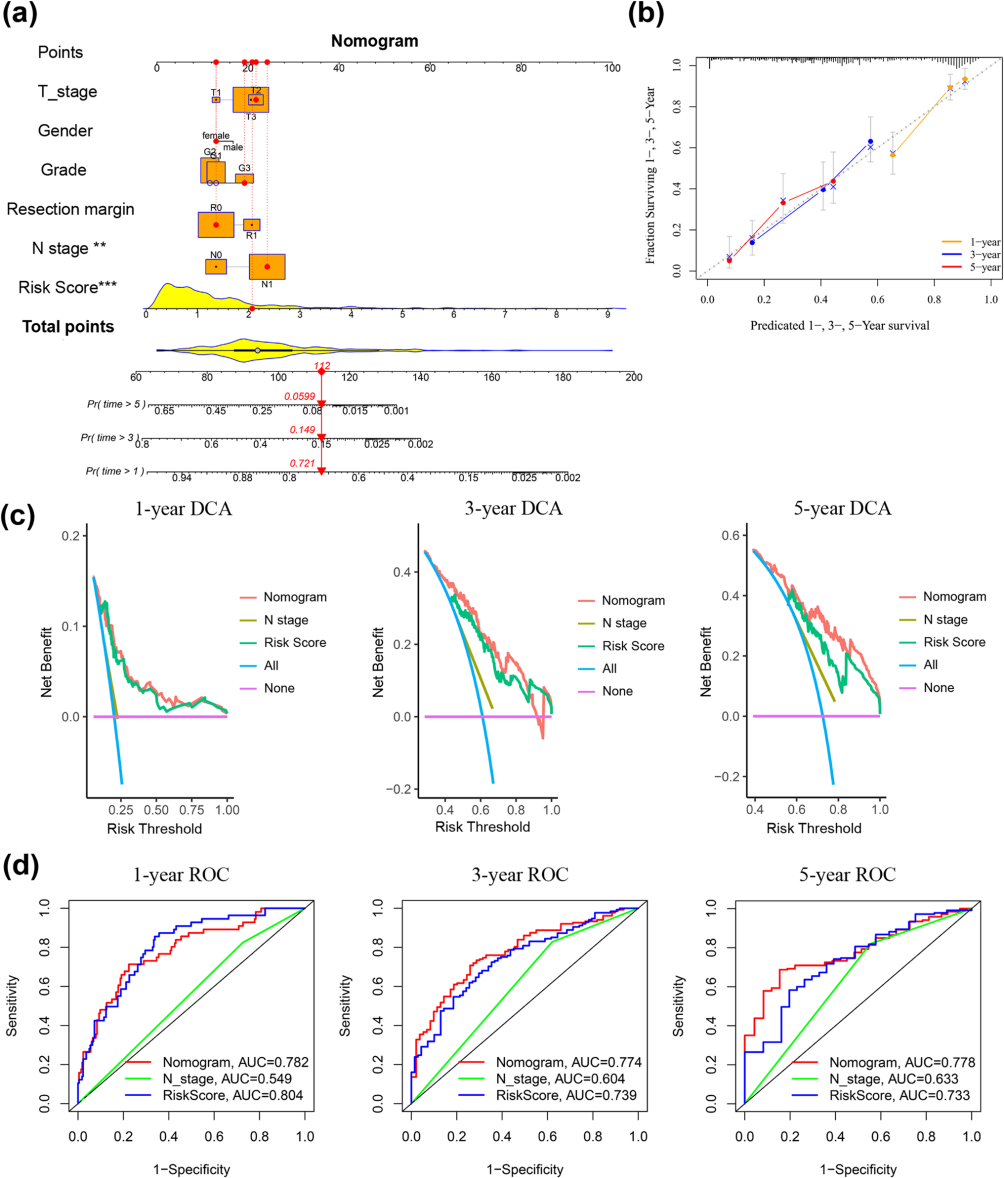
Establishment of the clinical nomogram. **a** Nomogram for predicting 1-, 3- and 5-year survival in PDAC patients. **b** The calibration curves of the nomogram. **c** The DCA curves of the nomogram compared for 1-, 3- and 5-year survival. **d** The ROC curves of the nomogram for 1-, 3- and 5-year survival. **p < 0.01, ***p < 0.001

To further predict the survival of PDAC patients, AJCC stage, resection margin, grade, gender and the ERS-related risk score were incorporated into a nomogram for predicting the 1-, 3- and 5-year survival rates (Fig. 6a). The calibration curve of the nomogram showed that the predicted 1-, 3- and 5-year survival outcomes were highly in accordance with the observed survival rate (Fig. 6b). Significantly, DCA was conducted to determine the clinical performance of the predictive nomogram, which showed that the nomogram provided a greater net benefit (Fig. 6c). Moreover, the nomogram was evaluated using ROC curves. As shown in Fig. 6d, the AUCs of nomogram at 1-, 3- and 5-year was 0.782, 0.774 and 0.778, which indicating well predictive performance of the nomogram.

### Function analysis of ERS-related signature

**Fig. 7:**
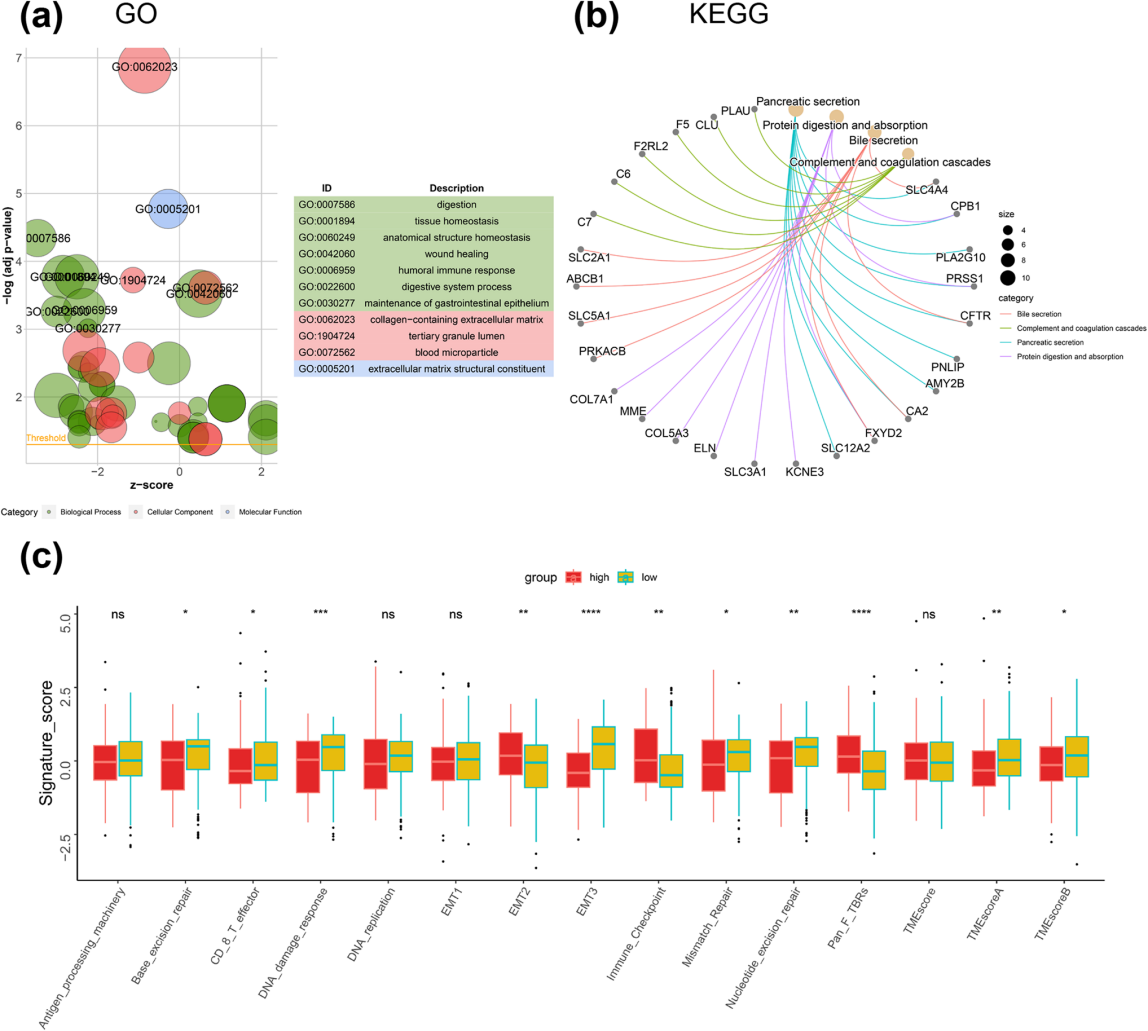
Functional enrichment analyses of ERS-related signature in PDAC. **a**, **b** GO (**a**) and KEGG (**b**) enrichment analyses between the two risk groups. **c** The expression of known signatures in high- and low-risk groups in E-MTAB-6134 cohort

**Fig. 8:**
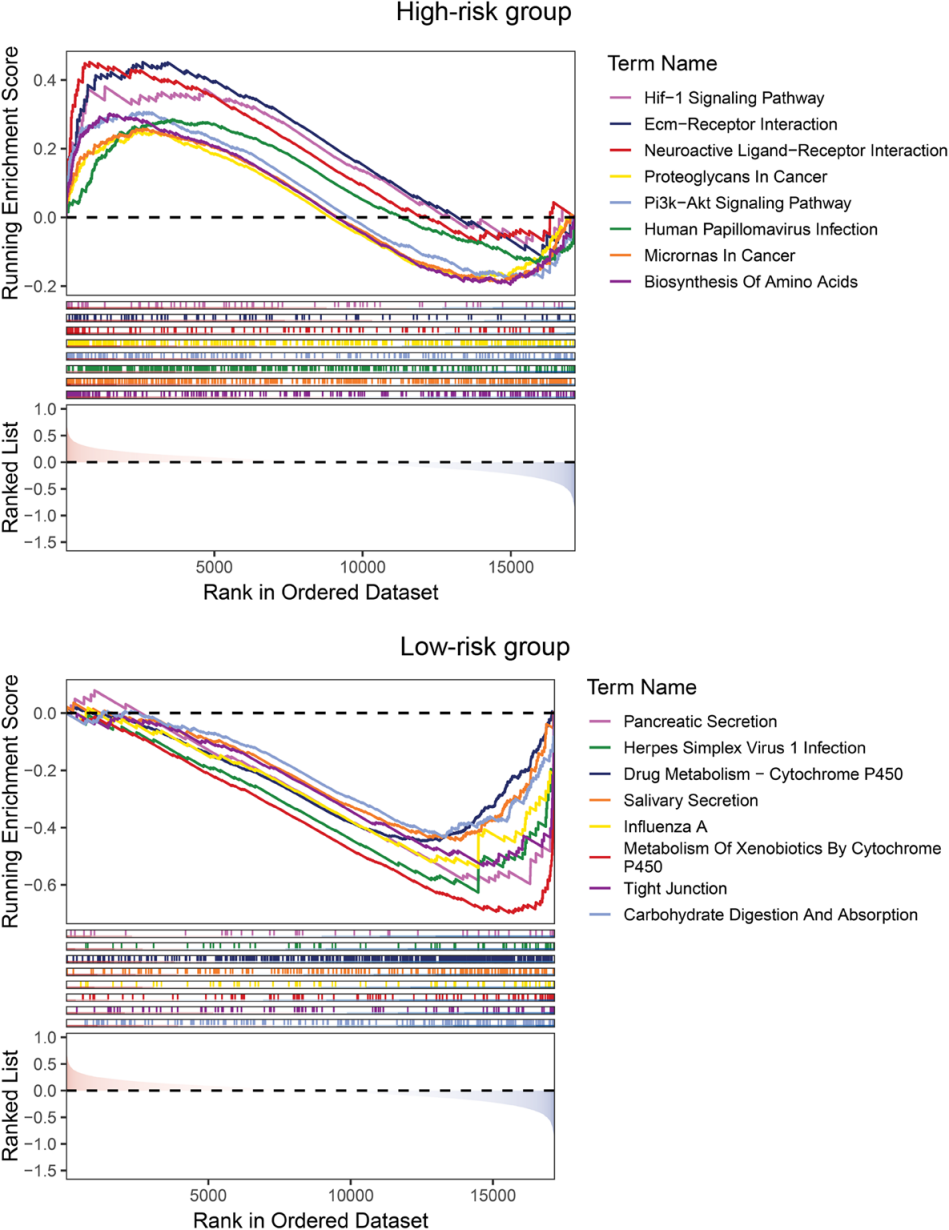
GSEA between the high-risk group (up) and low-risk group (down)

We identified DEGs between the high-risk group and low-risk group and performed functional enrichment analyses to further explore and confirm the signaling pathways enriched in the two groups. Based on the GO enrichment analysis, we found the top terms of Biological Process included digestion, tissue homeostasis, humoral immune response, digestive system process and maintenance of gastrointestinal epithelium. For Cellular Component, the top terms included collagen-containing extracellular matrix, tertiary granule lumen and blood microparticle. For Molecular Function, the extracellular matrix structural constituent term was enriched (Fig. 7a). Additionally, we discovered the bile secretion, complement and coagulation cascades, pancreatic secretion and protein digestion and absorption pathways were enriched based on KEGG enrichment analysis (Fig. 7b). Moreover, we investigated the correlation between the expression of ERS-related gene signature and the known signature and found that high-risk and low-risk PDAC patients in E-MTAB-6134 cohort are significantly different in base-excision repair, CD8^+^ T effector, DNA damage response, EMT2, EMT3, immune checkpoint, mismatch repair, nucleotide excision repair, Pan-F TBRs, TME scoreA and TME scoreB signature (Fig. 7c). In addition, the GSEA results revealed that the HIF-1 signaling pathway, ECM receptor interaction pathway and PI3K-AKT signaling pathway were enriched in the high-risk group, while the pancreatic secretion pathway, drug metabolism-cytochrome P450 pathway and tight junction pathway was enriched in the low-risk group (Fig. 8).

### Tumor immunity relevance of the ERS-related signature

**Fig. 9:**
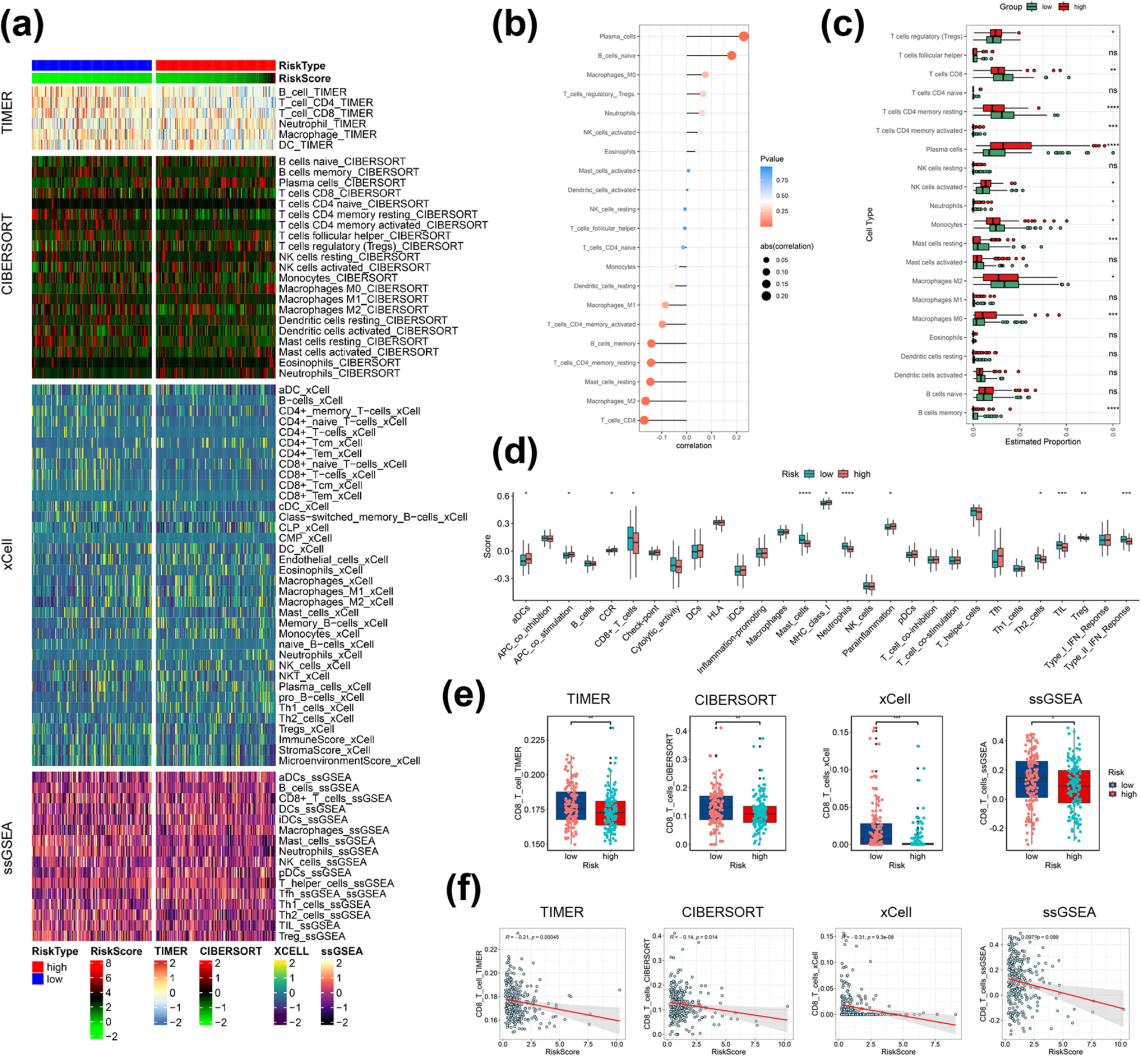
Correlation between immunity and the prognostic signature. **a** Immune cell infiltration landscape of the high- and low-risk groups of PDAC patients. **b** Correlation analysis of ERS-related risk score and immune infiltration. **c** The estimated proportion of 22 types infiltrated immune cell in two risk groups. **d** Immune function ssGSEA scores in the two risk groups. **e** The expression level of the CD8^+^ T cells in two risk groups calculated by TIMER, CIBERSORT, xCell and ssGSEA. **f** The correlation between CD8^+^ T cells and risk score analyzed by TIMER, CIBERSORT, xCell and ssGSEA. ns, not significant, *p < 0.05, **p < 0.01, ***p < 0.001

To verify the relationship between ERS-related prognostic signature and immune response, TIMER, CIBERSORT, xCell and ssGSEA algorithms were adopted to depict the landscape of infiltrating immune cells in PDAC patients (Fig. 9a). We also examined the correlation of ERS-related risk score and abundance of immune cells in PDAC tumor microenvironment via Spearman correlation analysis. Interestingly, CD8^+^ T cell infiltration had a negative correlation with risk score (Fig. 9b). Furthermore, in order to explore the infiltration of the 22 types of tumor-infiltrating immune cells in E-MTAB-6134 cohort, CIBERSORT algorithm was applied to estimate the levels of immune cell infiltration in each sample. As compared to high-risk group, patients in the low-risk group had higher level of infiltrated CD8^+^ T cells, CD4^+^ memory resting T cells, monocytes and M2 macrophages, while the infiltration levels of plasma cells, regulatory T cells, activated NK cells and M0 macrophages were significantly higher in the high-risk groups (Fig. 9c). In addition, as shown in Fig. 9d, we performed ssGSEA score comparison of immune function and results revealed that the APC costimulation, CCR, MHC class I and parainflammation pathways had higher enrichment scores in the high-risk group than in the low-risk, while the type II IFN response had a higher enrichment score in the low-risk group than that in the high-risk group. Importantly, in accordance with the evaluation of TIMER, CIBERSORT, xCell and ssGSEA algorithms, proportions of CD8^+^ T cells infiltrated in low-risk group were strongly higher than those in high-risk group (Fig. 9e, f).

**Fig. 10:**
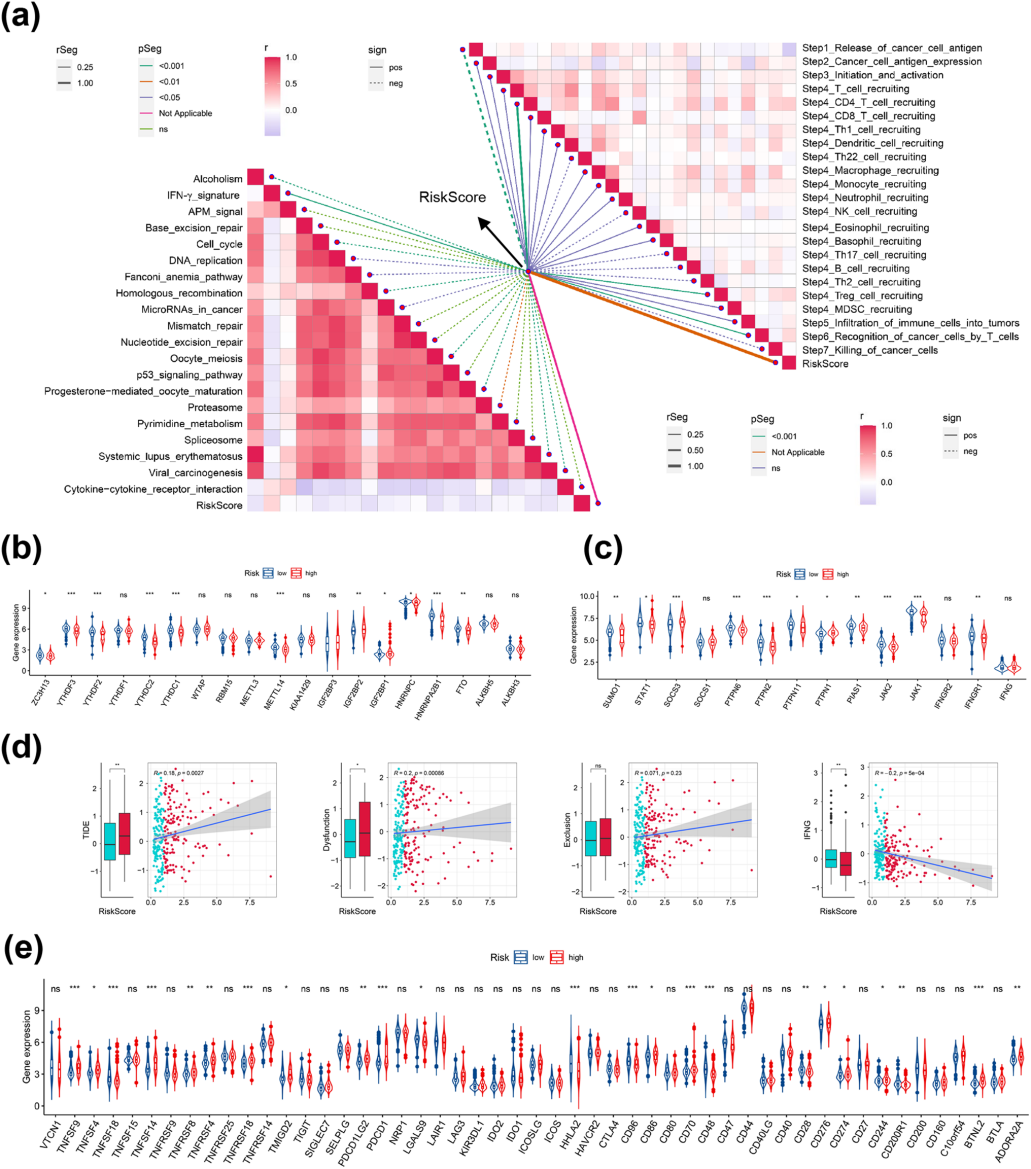
Prediction of immunotherapy response. **a** Correlations of ERS-related risk score with the activity of immunotherapy-predicted pathways and the steps of the cancer-immunity cycle. **b** The expression level of m6A regulator genes in two groups. **c** The expression level of IFN-γ pathway markers in two groups. **d** The pertinence between TIDE score and risk score and the TIDE score in two risk groups. **e** The expression level of immune checkpoint genes in two groups. ns, not significant, *p < 0.05, **p < 0.01, ***p < 0.001

Furthermore, we examined the differences in immune checkpoint blockade (ICB) response signature prediction between the two risk groups. The high-risk group scored highly in alcoholism, APM signal, cytokine-cytokine receptor interaction, homologous recombination, microRNAs in cancer, oocyte meiosis, progesterone-mediated oocyte maturation, proteasome, systemic lupus erythematosus and viral carcinogenesis, while scored lowly in IFN-γ signature than those in low-risk group (Fig. S6A). Moreover, except for IFN-γ signature, ERS-related risk score was negatively correlated with ICB-related positive signals (Fig. 10a). The tumor immune cycle is a critical indicator to assess the biological function of the chemokine system and other immunomodulators (Xu et al. 2018). Therefore, we evaluated the variations in the activity of tumor immune steps between two risk groups. As shown in Fig. S6B, high-risk group was highly scored in release of cancer cell antigen (step 1), while low-risk group highly scored in cancer cell antigen expression (step 2), CD4 T cell recruiting (step 4), Treg cell recruiting (step 4) and recognition of cancer cells by T cells (step 6). Similarly, we examined the correlation between risk score and these steps in tumor immune cycle. The results exhibited that risk score had a negative correlation with step 1, while positively correlated with the other tumor immune cycle steps (Fig. 10a). Considering the significant relevance between risk score and CD8^+^ T cells and the crucial role of m6A methylation in regulating the function of CD8^+^ T cells, we next investigated the expression of IFN-γ pathway markers and m6A regulators in two risk groups and most of them were remarkably associated with the ERS-related risk score (Fig. 10b, c). More importantly, to evaluate the immunotherapeutic efficacy between two risk groups, we calculated and compared the TIDE score. Our analysis suggested the risk score was positively correlate with TIDE score, indicating that low-risk PDAC patients may be more sensitive to immunotherapy than PDAC patients in high-risk group. Also, low-risk group had a higher IFNG score, while had a lowly score in the dysfunction (Fig. 10d). In addition, we evaluated the expression of immune checkpoints between the different risk groups. The results showed that immune suppressive molecules, such as PDCD1, TNFSF9, TNFSF4, TNFSF14, TNFRSF8, TNFRSF4, TNFRSF18, TMIGD2, PDCD1LG2, CD86, CD70, CD274 (PD-L1), CD276 (B7-H3), CD244, BTNL2 and ADORA2A, were elevated in the high-risk group, suggesting that patients in the high-risk group have a worse anti-tumor immune response (Fig. 10e). Also, we drawn a heatmap to exhibit the mRNA expression landscape of immune-modulator genes including chemokine, MHC, receptor, immune inhibitor and immune stimulator in two risk groups (Fig. S6C).

Taken together, these results indicated that the ERS-related signature had outstanding potential in evaluating the immunotherapy response. The unfavorable outcomes of high-risk PDAC patients might be due to an immunosuppressive microenvironment and a worse immunotherapy response.

### Correlation between risk score and drug sensitivity

**Fig. 11:**
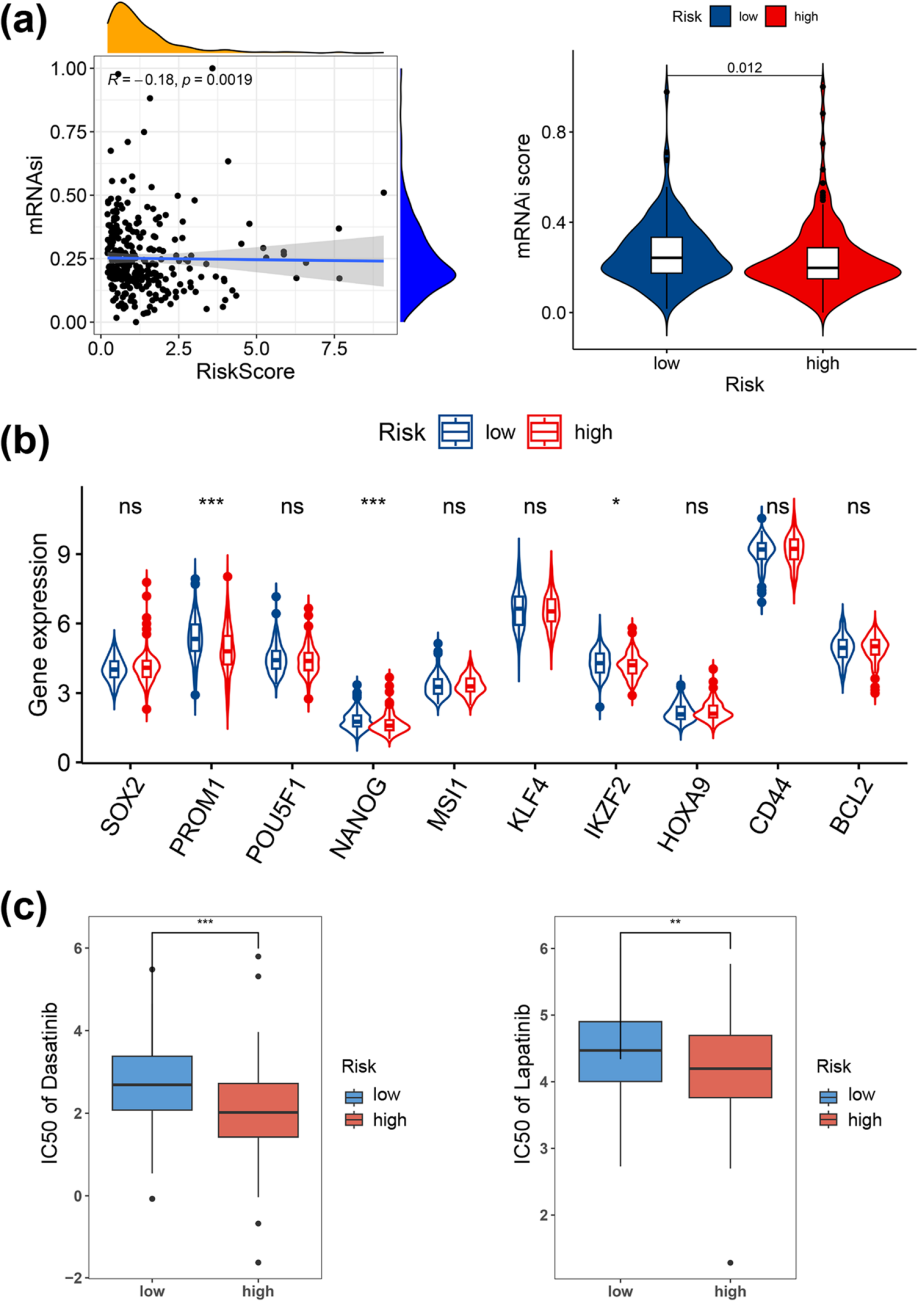
Prediction of chemotherapy response. **a** The pertinence between mRNAsi score and risk score and the mRNAsi score in two risk groups. **b** The expression level of stemness-related genes in two groups. **c** The IC50 of dasatinib and lapatinib in two groups. *p < 0.05, **p < 0.01, ***p < 0.001

Tumor stemness is known to play an important role in drug resistance. The mRNAsi is an index to describe the expression of stemness-associated genes of tumor cells and range from 0 to 1 (Malta et al. 2018). To investigate the correlation between the ERS-related risk score and drug sensitivity, mRNAsi score was evaluated. Our analysis shows mRNAsi score negatively correlates with risk score (R = − 0.18, *p* = 0.0019, Fig. 11a). We also specifically compared those stemness genes between the two risk groups. The stemness genes including PROM1, NANOG and IKZF2 were upregulated in low-risk group (Fig. 11b). Furthermore, based on the GDSC database, we predicted the response to common drugs among patients with PDAC by estimating the differences in the IC50 values between the high- and low-risk groups. Dasatinib, paclitaxel, BI.2536, gemcitabine, lapatinib and docetaxel (Zhu et al. 2022) were screened to assess the chemotherapy response of PDAC patients. Notably, the IC50 values of dasatinib and lapatinib in the high-risk group were significantly lower than those in the low-risk group, suggesting that patients in the high-risk group more sensitive to these two drugs (Fig. 11c).

### Experimental validation in vitro

**Fig. 12:**
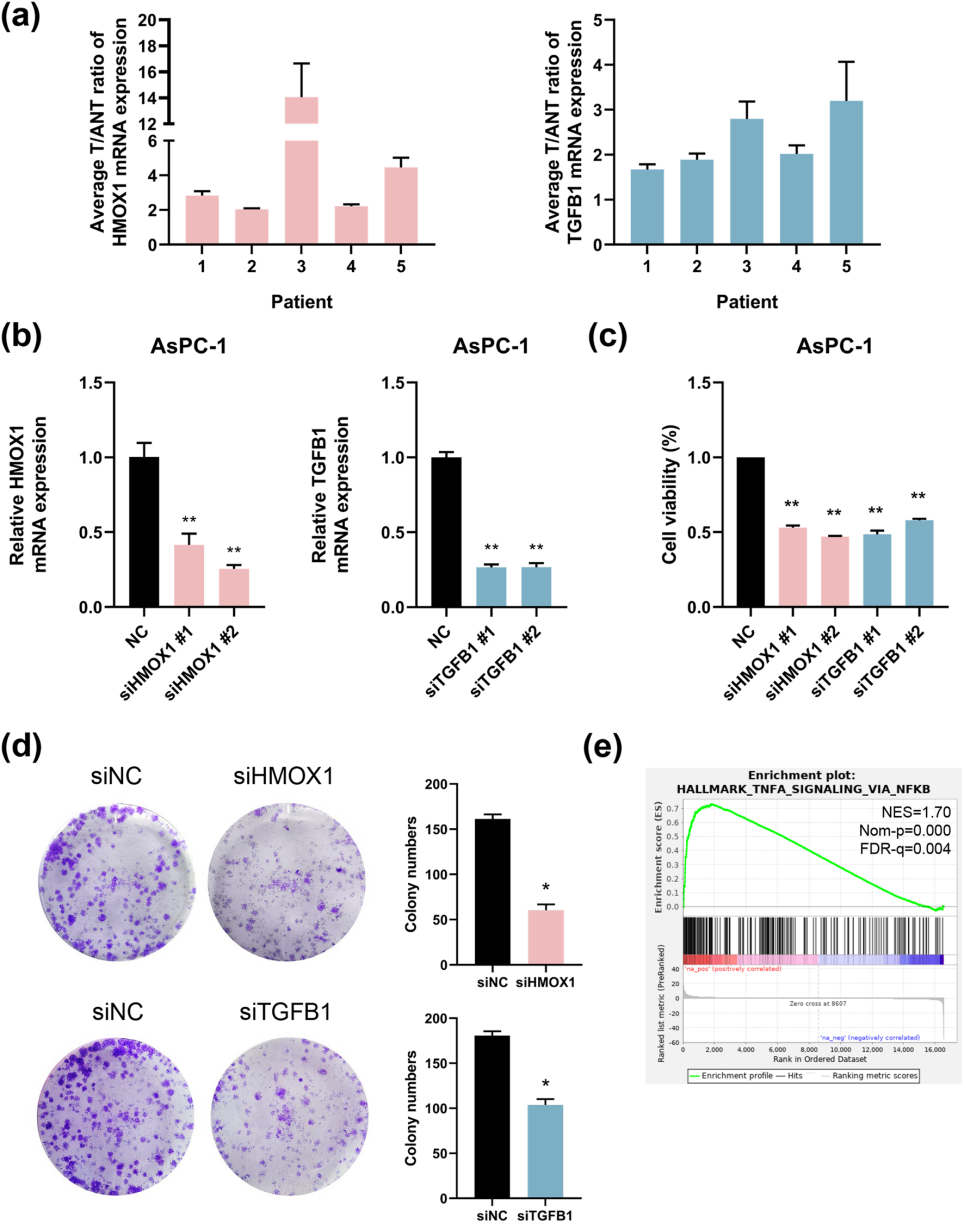
The impact of ERS-related genes on PDAC in vitro*.* **a** The average ratio of HMOX1 and TGFB1 expression in matched PDAC tissues (T) and adjacent nontumorous tissues (ANT). **b** The efficiency of siRNA-mediated knockdown in AsPC-1 cells. β-actin was included as a control. **c** CCK-8 assay was applied to detect the viability of AsPC-1 cells after silencing HMOX1 and TGFB1 at 48 h. **d** Effect of HMOX1 or TGFB1 knockdown on colony formation was measured in AsPC-1 cells. **e** GSEA analysis basing on the data of transcription sequencing identified HMOX1 expression level related signaling pathway in PDAC. Values are mean ± SD, *p < 0.05

To demonstrate the role of ERS-related genes in PDAC cells, we first analyzed the expression level in tumor and normal tissues of HMOX1 and TGFB1 using the TCGA, GSE41368 and GSE62165 datasets. As expected, HMOX1 and TGFB1 were upregulated in tumor and the related ROC curves showed the great performance of prediction (Fig. S7A, B). Furthermore, compared to the normal tissue, the HMOX1 and TGFB1 expression level in tumor tissue are mostly different in 33 tumor types (Fig. S7C). Then, we verified these findings by analyzing the FFPE tissues from PDAC patients. The results demonstrated that when compared to normal tissues, PDAC had higher levels of HMOX1 and TGFB1 expression (Fig. 12a). Moreover, siRNAs targeting HMOX1 and TGFB1 were transfected into AsPc-1 cells. The efficiency of siRNA knockdown was confirmed via q-PCR (Fig. 12b). As shown in Fig. 12c, compared to the control group, downregulation of HMOX1 and TGFB1 significantly suppressed the viability and of PDAC cells. Consistently, the results of clonogenic assays revealed that HMOX1 or TGFB1 knockdown reduced the potential for colony formation by tumor cells (Fig. 12d). To better understand what signaling pathway affects prognosis of PDAC, we conducted transcription sequencing of the AsPC-1 cells expressing RNAi against HMOX1 and scramble siRNA. Importantly, GSEA analysis revealed that TNF-α signaling via NF-κb pathway was notably enriched, which was worthy of further analysis (Fig. 12e). In summary, these findings suggest that ERS-related gene HMOX1 or TGFB1 may serve as the prognostic marker, which provided some new insights into predict the outcomes of PDAC patients.

## Discussion

PDAC is one of the most aggressive malignancies due to the lack of early diagnosis strategies and effective treatments. Despite advancements in surgical techniques and chemotherapy approaches, the survival benefits and methods of early diagnosis remain limited. Thus, searching for effective and novel biomarkers is critical for improving prognosis prediction among PDAC patients. Multiple studies have revealed that ERS are essential for cancer initiation and progression (Ferri and Kroemer 2001; Samanta et al. 2021). Considering the crucial roles of ERS in PDAC, in this study, we established a signature comprising eight ERS-related genes correlating with OS in PDAC. Subsequently, the eight ERS-related gene signature was demonstrated as independent prognostic factor by univariate, multivariate Cox and LASSO regression analysis. Moreover, a nomogram constructed with risk score could robustly predict the survival outcomes, which is beneficial for personalized treatment prefer to the TNM staging system.

In addition to functioning as predictors of patient survival, the genes in our signature also play crucial roles in the fundamental biology of PDAC and other cancers. Our study showed that reduction of HMOX1 and TGFB1 suppressed PDAC cell proliferation. It has been reported that HMOX1 upregulation enhances the growth and angiogenesis of pancreatic cancer during oxidative stress and HMOX1 downregulation improves responsiveness of PDAC cells to chemotherapy (Sunamura et al. 2003; Abdalla et al. 2019). On the other hand, recent studies have shown that enhanced HMOX1 expression leads to excessive accumulation of free cellular iron and severe lipid peroxidation, which augments ferroptosis and promotes the sensitivity of antitumor agents (Chang et al. 2018; Hassannia et al. 2018), suggested that the role of HMOX1 might be various in different tumor models. TGFB1 was demonstrated to mediate the regulation of malignant behaviors via activation of autophagy in PDAC and is involved in the development of chemoresistance (Abdel Mouti and Pauklin 2021). TGFB1 signaling also supports immunosuppression, promoting tumor development (Principe et al. 2016). GAPDH has been widely studied and deemed the hallmark of glycolysis in cancer metabolism. Methylated GAPDH was reported to suppress glycolysis in liver cancer cells and delay tumor cell proliferation (Zhong et al. 2018). A single-cell RNA sequencing study revealed that cancer-associated fibroblasts expressing CD74 could modulate the immune response in pancreatic tumors by activating CD4^+^ T cells (Elyada et al. 2019). CAV1 is a membrane protein associated with endocytosis, extracellular matrix organization, cholesterol distribution, and cell migration that has oncogenic functions via metabolic pathways (Dodson et al. 2013; Nwosu et al. 2016). Additionally, ERN2 was identified as a kinase controlling the quality of the ER and was found to play a significant role in patient survival (Southekal et al. 2021). CHRNE encodes the cholinergic receptor nicotinic epsilon sub unit and was reported associated positively with liver hepatocellular carcinoma prognosis (Zhang et al. 2022). Here, we found CHRNE negatively correlated with overall survival of PDAC patients and CHRNE knockdown inhibit the proliferation in PDAC cell line. These studies support the essential role of the ERS-related signature. Therefore, more basic experiments are required for further investigation.

Cancer immunotherapies have shown encouraging breakthroughs across multiple tumors; unfortunately, the response rate of immunotherapy for PDAC is disappointing (O'Reilly et al. 2019). Previous studies have revealed that ERS is highly correlated with immune cell infiltration, which regulates immune cell differentiation, activation, and cytokine expression (Smith 2018; Chen and Cubillos-Ruiz 2021). Tregs serve as key immunosuppressive cells in the setting of tumor immunity, while abundant cytotoxic CD8^+^ T cells are generally correlated with favorable prognosis and clinical response to immunotherapy (Sautes-Fridman et al. 2019; Nishikawa and Koyama 2021). In this study, we found that the low-risk group had a higher proportion of CD8^+^ T cells and a lower proportion of Tregs than the high-risk groups. This suggested that low-risk PDAC patients was more likely to benefit from immunotherapy. Meanwhile, we consistently found that checkpoints (PDCD1, CD274 and CD276) and the cytokines including TGFB1, IL-3, IL-4 and IL-10 involved in immune suppression were highly upregulated in high-risk group. Another concern is that inhibition or knockdown of PERK in melanoma cells made them hard to survive after ERS and resulted in immunogenic cell death (ICD) (Mandula et al. 2022), which exhibited the potential of targeting ERS in immunotherapy. Here, we investigated the immunotherapy response between the two risk groups and observed that PDAC patients with high ERS-related scores displayed higher TIDE scores. Additionally, we found the expression levels of key markers in two anti-tumor pathways associated with CD8^+^ T cells, IFN-γ pathway (St Paul and Ohashi 2020) and m6A pathway (Wang et al. 2020) in two risk groups indicating that the ERS-related signature was closely associated with the response to immunotherapy.

Targeted therapy is a crucial treatment strategy that can be used for treating PDAC (Qian et al. 2020; Singh and O'Reilly 2020). The currently available targeted drugs cannot be used for treating all PDAC patients as the therapeutic methods change with targetable subtypes of PDAC. Our study demonstrated that ERS-related signature is associated with the drug sensitivity of tyrosine kinase inhibitors dasatinib and lapatinib, which targets to tyrosine kinase SRC and EGFR/HER2 separately. PDAC patients in high-risk group might more sensitive to dasatinib and lapatinib treatments. In line with our study, human epidermal growth factor receptor 2 (HER2) has been identified as overexpressed in PDAC and related to poor patient survival (Komoto et al. 2009). Combination therapy with EGFR/HER2 inhibitor lapatinib could induce ERS and cause non‑canonical and lysosome‑dependent death in PDAC cells (Suzuki et al. 2022). Moreover, it has been reported that tyrosine kinase inhibitor dasatinib could suppress the transforming growth factor-beta-mediated promigratory responses in PDAC (Bartscht et al. 2015). Here, combine with our study, ERS-related signature could be used as a potential predictive biomarker in PDAC patients with dasatinib or lapatinib treatment. However, additional efforts are need to reveal the mechanism of ERS-related signature in PDAC chemotherapy.

In summary, our study revealed the value of ERS-related genes as prognostic biomarkers and verified that ERS plays a crucial role in PDAC progression. Based on the data of transcription sequencing, GSEA analysis revealed that TNF-α signaling via NF-κb pathway was differentially enriched. However, further research is required. Besides, this study still has some inevitable limitations. For example, we performed an analysis on retrospective data obtained from a public database that did not contain all the information about the PDAC cases. Conducting prospective clinical studies in multiple centers would produce robust prognostic risk models. In addition, more relevant experiments and clinical evidence are needed to further confirm the biological mechanisms of these prognostic ERS-related genes.

## Conclusion

In conclusion, we established and validated an ERS-related signature for predicting the prognosis of PDAC patients. Further studies suggested that the signature could be used for predicting immunotherapy response and potential anti-tumor drug sensitivity of PDAC patients, which could be beneficial for making the clinical strategies and treating.

## Supplementary Information

Below is the link to the electronic supplementary material.

Figure S1 Functional enrichment analysis of ERS-related DEGs. (A) GO enrichment analysis. (B) KEGG enrichment analysis (TIF 3757 KB)

Figure S2 Univariate analysis with Cox proportional hazard model (TIF 634 KB)

Figure S3 Association between the ERS-related signature and the outcome of PDAC. (A) Principal components analysis. (B) Heatmap displayed the expression of the 8 prognostic genes in high- and low-groups in E-MTAB-6134, TCGA, GSE21501, GSE28735 and GSE62452 (TIF 6427 KB)

Figure S4 Evaluation and validation of the ERS-related prognostic signature in validation cohorts of PDAC(A) Kaplan–Meier survival analysis of PDAC patients between high- and low-risk groups. (B) Distribution of survival status based on the median risk score of PDAC patients. (C) ROC curves to predict the sensitivity and specificity of 1-, 3- and 5-year survival according to the ERS-related signature in GSE62452, while the sensitivity of 1-, 2- and 3-year survival were validated in GSE28735 (TIF 931 KB)

Figure S5 Kaplan–Meier survival analysis of risk score groups in indicated clinicopathological subgroups (TIF 806 KB)

Figure S6 Roles of ERS-related signature in predicting immune phenotypes in the E-MTAB-6134 cohort. (A) The disparity of immunotherapy prediction pathway in enrichment scores between high- and low-risk groups. (B) The disparity of each step of cancer-immunity cycle in enrichment scores between high- and low-risk groups. (C) Heatmap displayed the vary mRNA expression of immunomodulators including chemokine, MHC, receptor, immune inhibitor and immune stimulator. ns, not significant, * p<0.05, ** p<0.01, *** p<0.001 (TIF 5631 KB)

Figure S7 The expression level of HMOX1 and TGFB1. (A) The expression level of HMOX1 and TGFB1 in PDAC and normal tissues between high- and low-risk group from TCGA, GSE41368 and GSE62165 datasets. (B) The ROC curves showed the predictive sensitivity and specificity of the HMOX1 and TGFB1 expression. (C) The expression level of HMOX1 and TGFB1 in normal and tumor tissues in 33 tumor types. * p<0.05, ** p<0.01, *** p<0.001 (TIF 1773 KB)

Table S1 163 DEGs from the intersection of E-MTAB-6134 and TCGA (XLSX 19 KB)

Table S2 GSEA (XLS 41 KB)

Table S3 The siRNA sequence used in the study (XLS 26 KB)

Table S4 The qPCR primer pairs used in the study (XLS 25 KB)

## Data Availability

The data generated during the current study are available from the corresponding author on reasonable request.
