# Supplementary figures and images for: An endoplasmic reticulum stress-related signature could robustly predict prognosis and closely associate with response to immunotherapy in pancreatic ductal adenocarcinoma
Source: J Cancer Res Clin Oncol. 2023 Aug 31;149(17):15589–608. doi: 10.1007/s00432-023-05312-x (PMC10620278; doi:10.1007/s00432-023-05312-x)

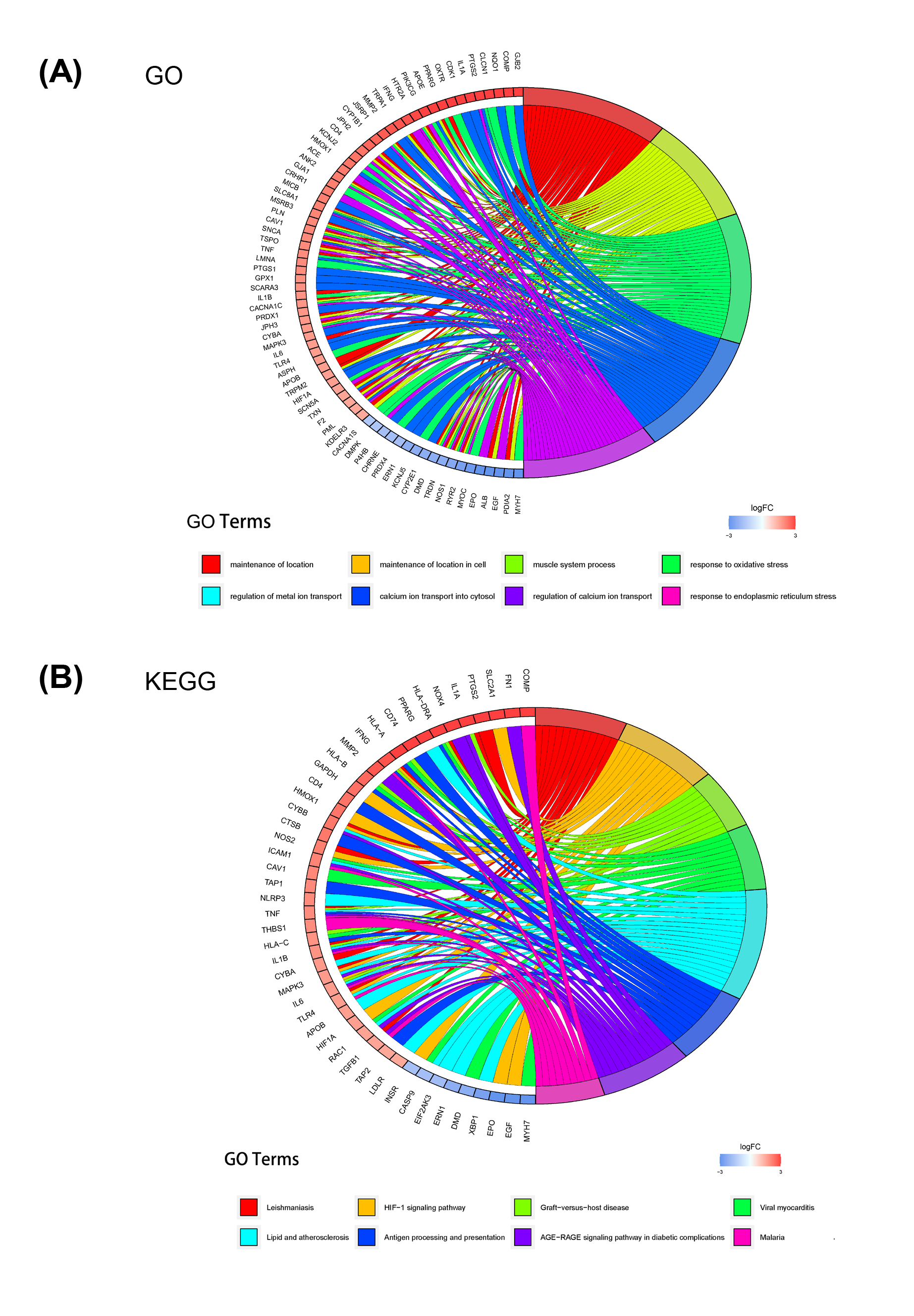

Supplement: Supplementary file 1 — Figure S1 Functional enrichment analysis of ERS-related DEGs. (A) GO enrichment analysis. (B) KEGG enrichment analysis (TIF 3757 KB) [file 432_2023_5312_MOESM1_ESM.tif]

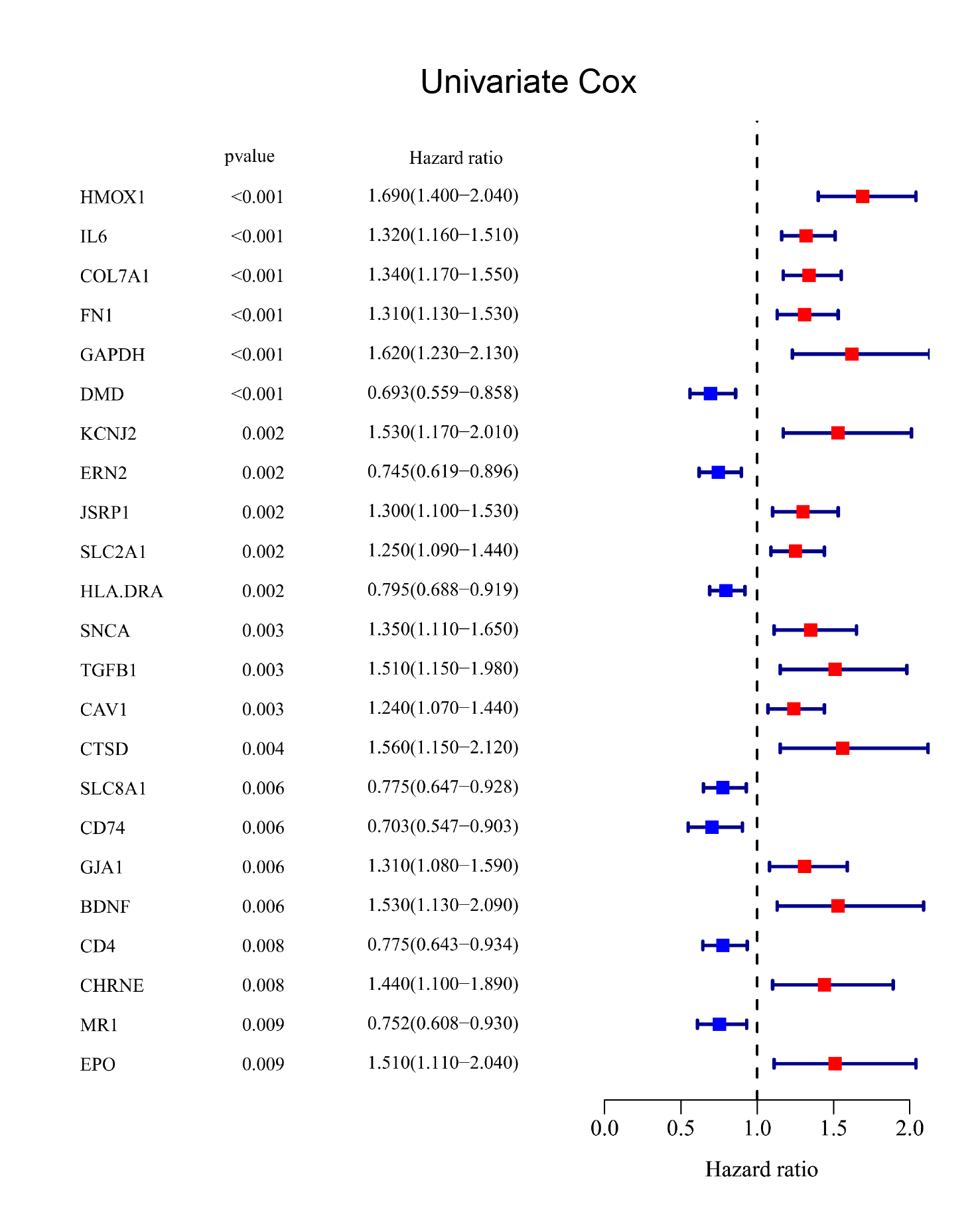

Supplement: Supplementary file 2 — Figure S2 Univariate analysis with Cox proportional hazard model (TIF 634 KB) [file 432_2023_5312_MOESM2_ESM.tif]

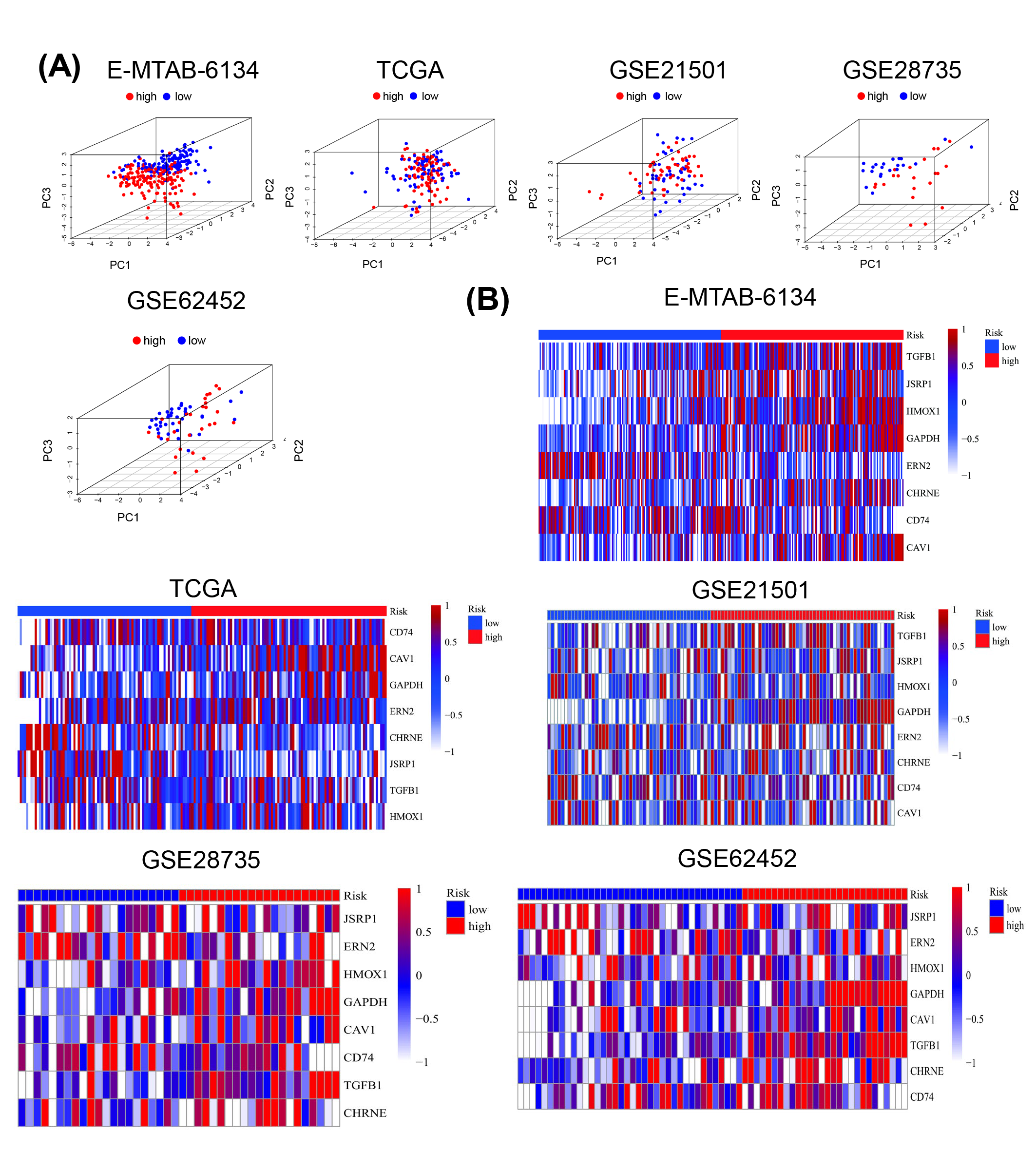

Supplement: Supplementary file 3 — Figure S3 Association between the ERS-related signature and the outcome of PDAC. (A) Principal components analysis. (B) Heatmap displayed the expression of the 8 prognostic genes in high- and low-groups in E-MTAB-6134, TCGA, GSE21501, GSE28735 and GSE62452 (TIF 6427 KB) [file 432_2023_5312_MOESM3_ESM.tif]

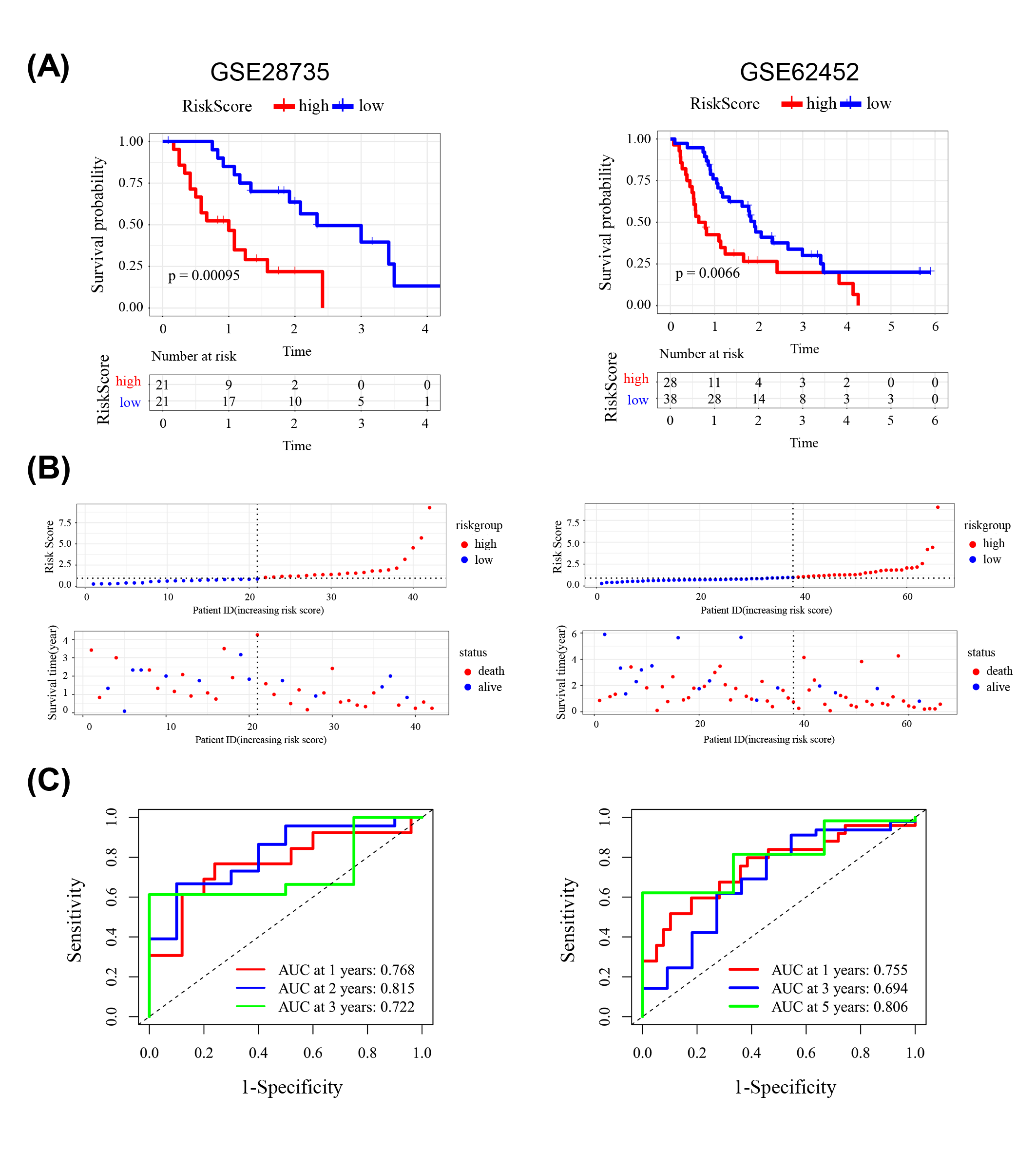

Supplement: Supplementary file 4 — Figure S4 Evaluation and validation of the ERS-related prognostic signature in validation cohorts of PDAC(A) Kaplan–Meier survival analysis of PDAC patients between high- and low-risk groups. (B) Distribution of survival status based on the median risk score of PDAC patients. (C) ROC curves to predict the sensitivity and specificity of 1-, 3- and 5-year survival according to the ERS-related signature in GSE62452, while the sensitivity of 1-, 2- and 3-year survival were validated in GSE28735 (TIF 931 KB) [file 432_2023_5312_MOESM4_ESM.tif]

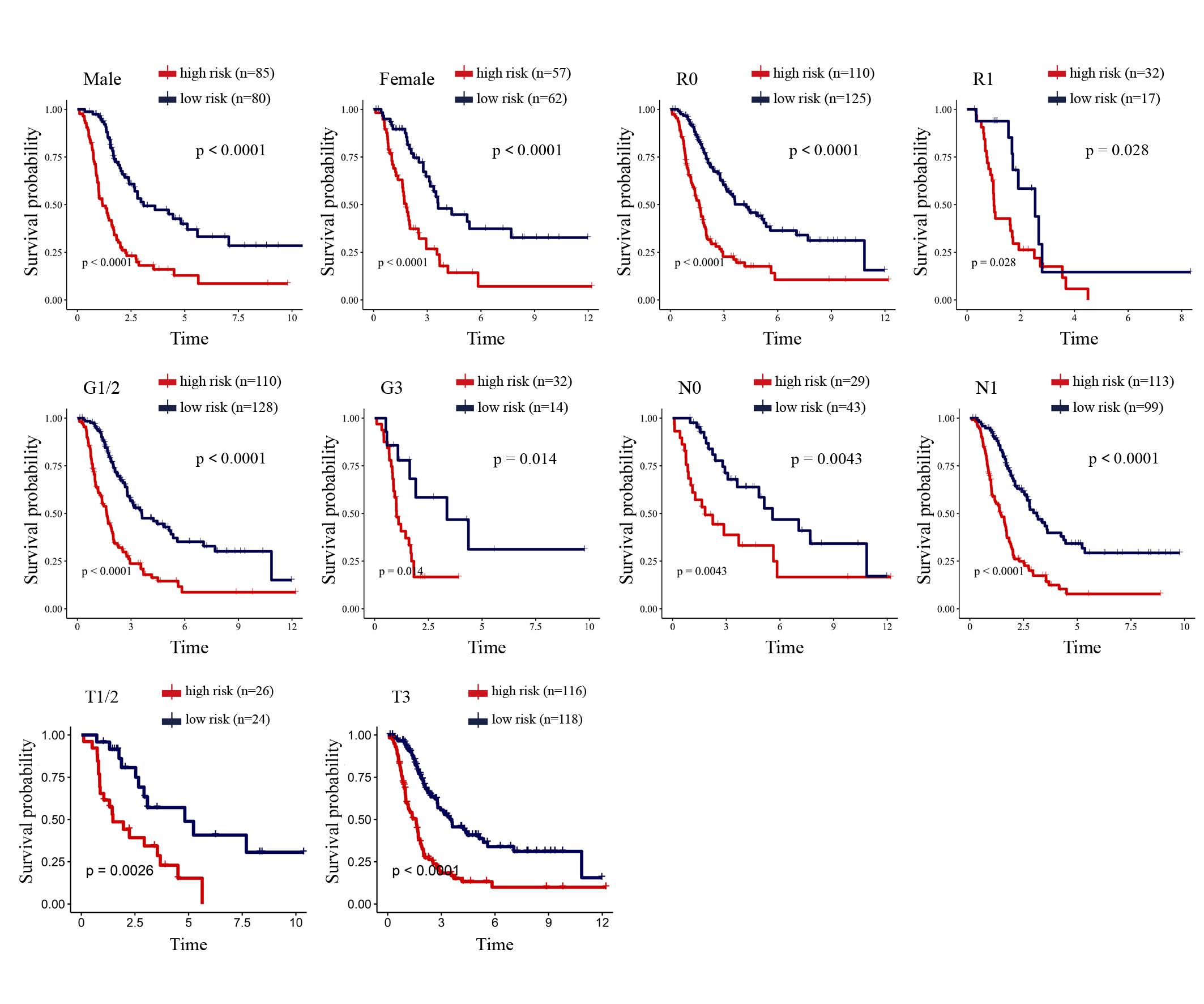

Supplement: Supplementary file 5 — Figure S5 Kaplan–Meier survival analysis of risk score groups in indicated clinicopathological subgroups (TIF 806 KB) [file 432_2023_5312_MOESM5_ESM.tif]

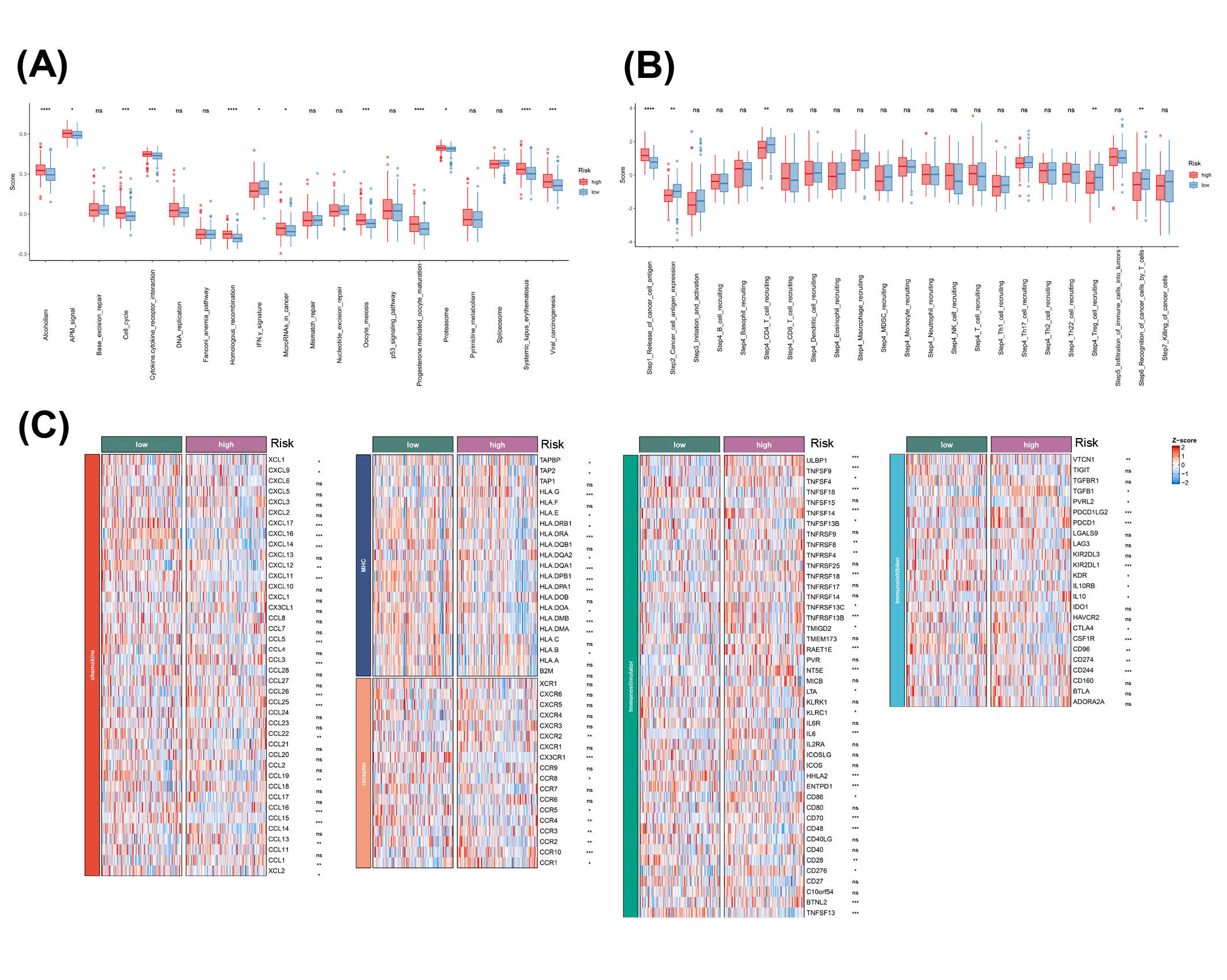

Supplement: Supplementary file 6 — Figure S6 Roles of ERS-related signature in predicting immune phenotypes in the E-MTAB-6134 cohort. (A) The disparity of immunotherapy prediction pathway in enrichment scores between high- and low-risk groups. (B) The disparity of each step of cancer-immunity cycle in enrichment scores between high- and low-risk groups. (C) Heatmap displayed the vary mRNA expression of immunomodulators including chemokine, MHC, receptor, immune inhibitor and immune stimulator. ns, not significant, * p<0.05, ** p<0.01, *** p<0.001 (TIF 5631 KB) [file 432_2023_5312_MOESM6_ESM.tif]

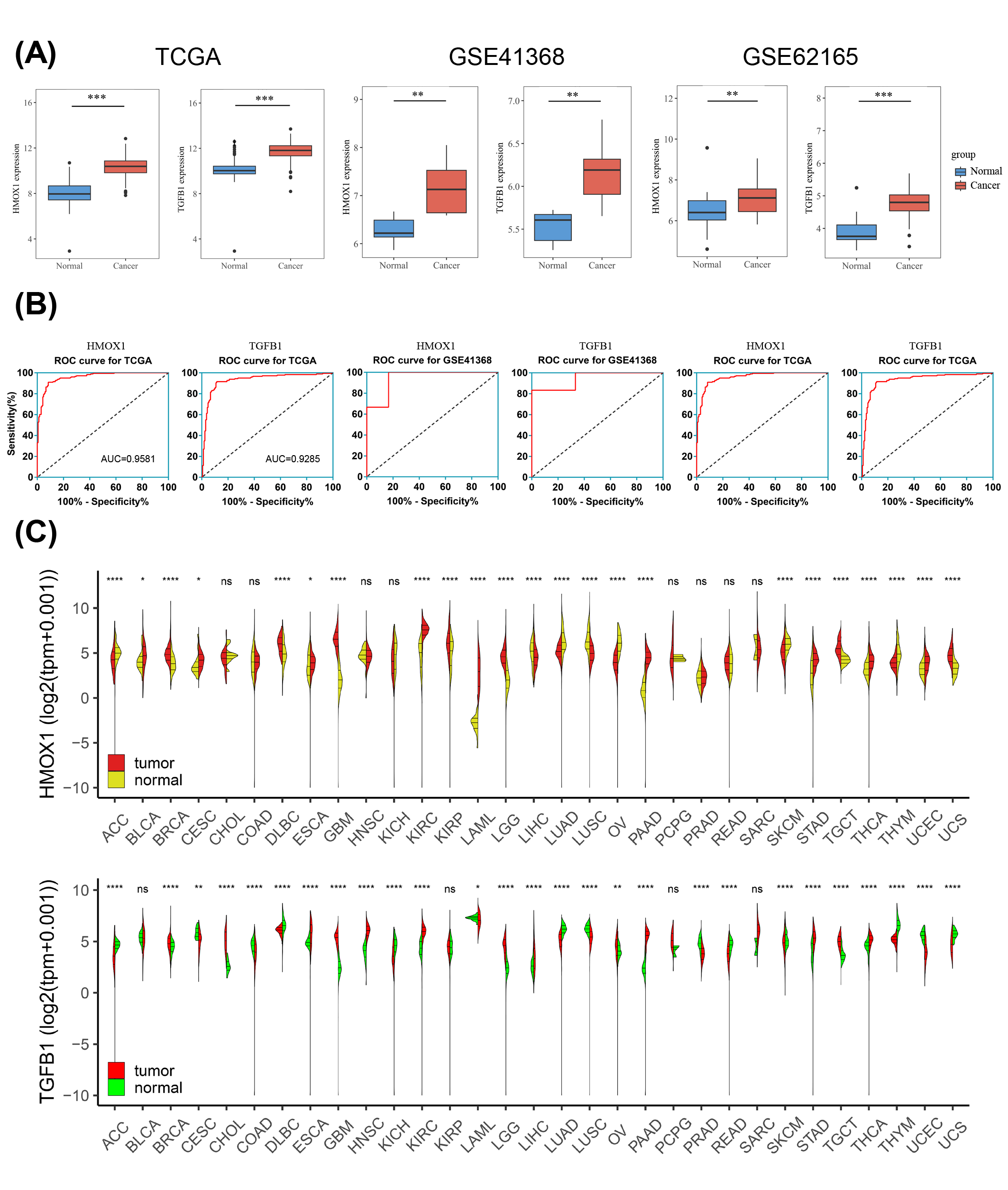

Supplement: Supplementary file 7 — Figure S7 The expression level of HMOX1 and TGFB1. (A) The expression level of HMOX1 and TGFB1 in PDAC and normal tissues between high- and low-risk group from TCGA, GSE41368 and GSE62165 datasets. (B) The ROC curves showed the predictive sensitivity and specificity of the HMOX1 and TGFB1 expression. (C) The expression level of HMOX1 and TGFB1 in normal and tumor tissues in 33 tumor types. * p<0.05, ** p<0.01, *** p<0.001 (TIF 1773 KB) [file 432_2023_5312_MOESM7_ESM.tif]
